# Parental childhood vaccine hesitancy and predicting uptake of vaccinations: a systematic review

**DOI:** 10.1017/S1463423622000512

**Published:** 2022-11-04

**Authors:** Kennedy Obohwemu, Floor Christie-de Jong, Jonathan Ling

**Affiliations:** University of Sunderland, Sunderland, UK

**Keywords:** childhood vaccines, confidence, immunisation, hesitancy, parents, public trust, vaccination, vaccine

## Abstract

**Aim:**

This review aims are to (1) identify relevant quantitative research on parental childhood vaccine hesitancy with vaccine uptake and vaccination intention being relevant outcomes and (2) map the gaps in knowledge on vaccine hesitancy to develop suggestions for further research and to guide interventions in this field.

**Background:**

Vaccine hesitancy recognises a continuum between vaccine acceptance and vaccine refusal, de-polarising past anti-vaccine, and pro-vaccine categorisations of individuals and groups. Vaccine hesitancy poses a serious challenge to international efforts to lessen the burden of vaccine-preventable diseases. Potential vaccination barriers must be identified to inform initiatives aimed at increasing vaccine awareness, acceptance, and uptake.

**Methods:**

Five databases were searched for peer-reviewed articles published between 1998 and 2020 in the fields of medicine, nursing, public health, biological sciences, and social sciences. Across these datasets, a comprehensive search technique was used to identify multiple variables of public trust, confidence, and hesitancy about vaccines. Using PRISMA guidelines, 34 papers were included so long as they focused on childhood immunisations, employed multivariate analysis, and were published during the time frame. Significant challenges to vaccine uptake or intention were identified in these studies. Barriers to vaccination for the target populations were grouped using conceptual frameworks based on the Protection Motivation Theory and the World Health Organization’s Strategic Advisory Group of Experts on Immunization Working Group model and explored using the 5C psychological antecedents of vaccination.

**Findings:**

Although several characteristics were shown to relate to vaccine hesitancy, they do not allow for a thorough classification or proof of their individual and comparative level of influence. Understudied themes were also discovered during the review. Lack of confidence, complacency, constraints, calculation, and collective responsibility have all been highlighted as barriers to vaccination uptake among parents to different degrees.

## Introduction

Apart from the provision of clean water, vaccines have had a more profound effect on global health, especially children, than any other public health measure (Public Health England, 2014; WHO, 2021; Rodrigues & Plotkin, 2020). Despite this, millions of children around the world do not receive the recommended vaccines. In 2020, 23 million children missed out on routine childhood vaccinations, the highest number since 2009 and 3.7 million higher than in 2019 (WHO, 2021; UNICEF, 2022).

Poor vaccination coverage leads to outbreak of diseases (UNICEF, 2019). For example, in January and February 2022, there were over 17 338 cases of measles recorded globally, compared to 9665 cases in the same period in 2021 (WHO, 2022). In England and Wales in 2018, there was a marked increase in confirmed measles cases with 991 cases, compared to 284 cases in 2017 (Public Health England, 2019). These developments led to the UK losing its ‘measles-free’ status with the World Health Organization (WHO) barely three years after the measles virus was eliminated from the country (Wise, 2019).

Concern from parents, decision-makers, and the media regarding the safety of recommended immunisations has increased in recent years due to debates regarding the links between vaccines and autism, vaccine ingredients, and the number of injections given during a single office visit or during the first years of life (Miller & Reynolds, 2009; Davidson, 2022; Gabis *et al.*, 2022). An increasing number of people question the safety of vaccines (Yaqub *et al.*, 2014; Dubé, *et al.* 2015; Larson *et al.*, 2015a), seek alternative measures such as natural methods (eg, rigorous hygiene) and antibiotic use (Dempsey *et al.*, 2011; Robison *et al.*, 2012; Popa *et al.*, 2020) and sometimes delay or refuse vaccination (Gust *et al.*, 2008; Larson *et al.*, 2014a; Larson *et al.*, 2014b). This delay or refusal of vaccination is termed vaccine hesitancy (VH). VH is of grave concern, such that it was listed by the WHO as one of the ten threats to global health in 2019 (WHO, 2019).

VH is determined by a wide range of factors. In the UK, a cross-sectional study of 600 participants including GPs, health visitors, practice nurses and parents of immunised children found that socioeconomic factors, such as high social class and being a first-time parent, were important predictors of delayed childhood vaccination (Macdonald *et al.*, 2004). Family size and parental education were identified as determinants of under-immunisation in Greece (Danis *et al.*, 2010). In Nigeria, maternal availability, lack of knowledge and parental disapproval were associated with partial immunisation (Babalola, 2011). A combination of sociodemographic and socioeconomic factors such as marital status, maternal education and family income influenced parental decision-making in Israel (Stein-Zamir & Israeli, 2017), Saudi Arabia (Alsubaie *et al.*, 2019), Italy (Giambi *et al.*, 2018), Australia (Chow *et al.*, 2017), and USA (Omer *et al.*, 2009; Rachel *et al.*, 2018).

Several systematic reviews have investigated factors that influence VH across different populations, with a particular focus on the influence of knowledge, attitudes, and beliefs on vaccination behaviour (Falagas & Zarkadoulia, 2008; Rainey *et al.*, 2011; Prematungr *et al.*, 2012; Trim *et al.*, 2012). While it is important to identify potential determinants of VH, the proportion of parents who are vaccine-hesitant needs to be estimated using widely validated, theory-based psychological scales, to inform researchers and policymakers about the burden of vaccine-preventable diseases (VPDs), which will ultimately help in identifying priorities in healthcare prevention, promotion, practices, and policy (Bloom, 2008; Mahase, 2020). Few studies offer quantitative tools to measure prevalence of VH and even fewer studies have used standardised, widely validated survey instruments, such as the Parent Attitudes about Childhood Vaccinations scale (Opel *et al.,* 2011a; Opel *et al.*, 2011b), to achieve these objectives.

**Fig. 1.**
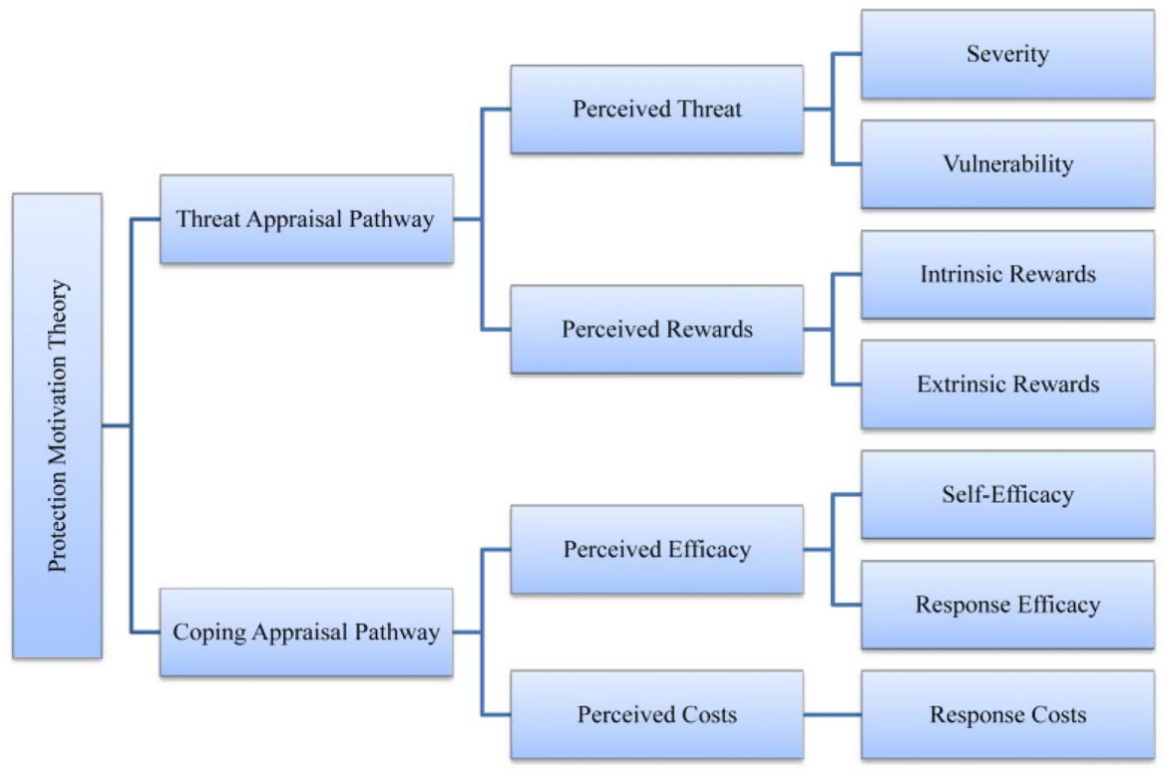
PMT constructs

Few researchers have applied theories of health behaviour to vaccination uptake. The Protection Motivation Theory (PMT) (Rogers, 1975; Rogers, 1983; Maddux & Rogers, 1983; Rogers & Prentice-Dunn, 1997), developed to understand how people respond to health threats, is one such theory. PMT suggests that people will be likely to protect themselves (eg, by obtaining a vaccine) if they have firm beliefs about the threat posed by the disease itself (severity and vulnerability) (Voeten *et al.*, 2009). PMT considers the physical and psychosocial consequences of engaging in a risky behaviour (intrinsic and extrinsic rewards) and the costs (eg, personal resources) involved in avoiding the given health threat (response costs) (Rogers & Prentice-Dunn, 1997), as displayed in Figure 1. In addition, PMT considers people’s beliefs in their own abilities to adopt a protective measure (self-efficacy) as well as the outcomes of their behaviour (response efficacy) (Maddux & Rogers, 1983; Rogers & Prentice-Dunn, 1997). PMT thus reliably predicts behavioural intentions based on attitudes and perceptions (de Zwart *et al.*, 2009).

**Fig. 2.**
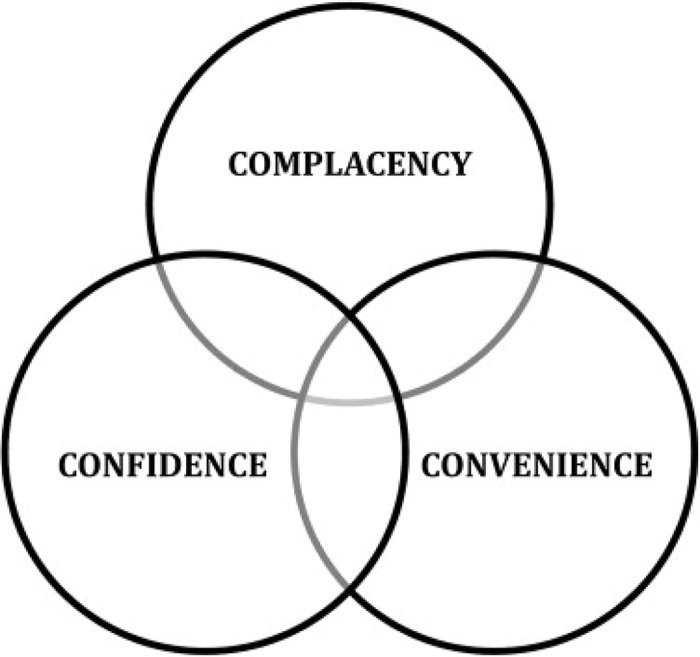
3C Model of vaccine hesitancy

Vaccination acceptance is a behavioural outcome that results from a complicated decision-making process that can be impacted by a variety of variables. After considering the diverse factors and the possibility of informing the development of global and country-level VH indicators, the WHO’s Strategic Advisory Group of Experts on Immunization (SAGE) Working Group developed the 3C model of VH (MacDonald, 2015), which points out three different types of VH determinants: confidence, complacency, and convenience (Figure 2).

*Confidence* is described in the 3C model as strong belief in the efficacy and safety of vaccinations, the system that distributes them, plus the trustworthiness and competency of health services, health systems and health professionals, and what drives the policymakers who determine which vaccines are required (MacDonald, 2015). People with little or no vaccine confidence have negative views toward immunisations, which influence their actions. The unfavourable attitude is fuelled by misinformation, conspiracy theories, and heightened perceptions of vaccine-related risks (Betsch *et al.*, 2015).

*Complacency* occurs when the dangers of VPDs are viewed as minimal, and vaccination is not considered a required precautionary measure (MacDonald, 2015). Complacency about a specific vaccine, or vaccination in general, is determined by several factors, which include other general duties that are deemed more important at the time (Betsch *et al.*, 2015). When people assess the risks of getting a specific vaccine versus the risks of getting the disease that the vaccine protects against, the success of vaccination programmes can lead to complacency and, ultimately, hesitancy (Schmid *et al.*, 2017). The extent to which complacency affects hesitancy is also determined by self-efficacy (an individual’s perceived or real ability to decide whether to be vaccinated or not) (Ernsting *et al.*, 2015).

*Convenience* is a crucial determinant which might result from sentiments that are neither strongly against nor strongly in favour of vaccination, implying that vaccination is insufficiently important to actively overcome physical or psychosocial barriers (MacDonald, 2015). For example, access to immunisations may be hampered by geopolitical or economic reasons that affect production and supply reliability (Betsch *et al.*, 2015). Furthermore, increased vaccine costs may result in a reduction in the frequency with which parents interact with healthcare services (Schmid *et al.*, 2017). As a result, when decision-makers face obstacles such as poor access, a high cost, or a long travel time, they opt out of vaccination to avoid these issues (Betsch *et al.*, 2015).

Grounded in the 3C model and other validated VH and acceptance models (Larson *et al.*, 2014a; Thomson *et al.*, 2016), the 5C model captures relevant determinants of vaccination behaviour and links them to psychological theories to explain health behaviour (Betsch *et al.*, 2018). The 5C model retains the terms ‘confidence’ and ‘complacency’, but ‘convenience’ is replaced with ‘constraints’ as it more accurately reflects the physical, structural, and psychological obstacles (eg, access, costs) that serve as gatekeepers, preventing the transition from vaccination intention to vaccination behaviour. Time spent travelling to vaccination centres or enduring unpleasant procedures can also be categorised as *constraints* (Betsch *et al.*, 2015).

*Calculation*, the fourth ‘C’ which applies to both the 4C (Betsch *et al.*, 2015) and 5C models (Betsch *et al.*, 2018), demonstrates the requirement for significant research and elaboration. People with high calculation tendencies assess the risks of infection and immunisation to make an informed decision. As a result, calculation has been linked with the risk of disease exposure and immunisation (Brewer *et al.*, 2007). Cost-benefit analysis could indicate a risk-averse mindset, hence a negative correlation with risk attitude (Johnson *et al.*, 2004). The need to avoid risks could be a major incentive to people with high calculation levels, as their conscious thinking patterns suggest (Johnson *et al.*, 2004). These individuals are also known to have a more deliberate logical and cognitive decision-making style (Betsch *et al.,* 2015) and to rely less on superstitious beliefs (Wiseman & Watt, 2004).

*Collective responsibility* refers to a person’s willingness to safeguard others through herd immunity (Fine *et al.*, 2011). The notion includes the societal benefits of vaccination, such as the fact that most immunisations protect unvaccinated individuals owing to herd immunity. The desire to free-ride when enough people are vaccinated is the opposite effect (Fine *et al.*, 2011; Betsch *et al.*, 2013; Betsch *et al.*, 2017). Collectivism, communal attitude, and empathy have been associated with collective responsibility (Clark *et al.*, 1987; Shulruf *et al.*, 2007; Betsch *et al.*, 2017). Because collective responsibility has a negative correlation with individualism (Shulruf *et al.*, 2007), those with a high sense of collective responsibility are likely to vaccinate in the interests of others. Low levels may suggest that a person is unaware of herd immunity, is unconcerned about it, or refuses to vaccinate in the interest of others (Betsch *et al.*, 2015).

Examining psychological variables is critical for understanding vaccination intention and informing effective interventions (Schmid *et al.*, 2017). A more comprehensive knowledge and understanding of the underlying psychology of vaccine-hesitant groups can improve the effectiveness of public health messages aimed at these populations.

This systematic review uses the PMT and the WHO’s SAGE Working Group model as comprehensive theoretical frameworks for understanding VH and its drivers. The models served as useful tools for predicting the intention of parents to adopt protective behaviours, such as getting their children vaccinated. The physical, psychological, contextual, and sociodemographic barriers to vaccination will be identified and clustered using these theoretical models. The hesitancy profiles of the identified risk group (parents) were discussed using the 5C model, and the findings were integrated at the macro- and micro-level.

This paper examined VH from a global perspective and then narrowed its focus to the UK. The purpose is to understand parental childhood VH and inform gaps in research and interventions in the UK and, importantly, consider the wider determinants of VH as no single intervention exists to eliminate VH (WHO, 2021; Danabal *et al.*, 2021; Wiysonge *et al.*, 2022).

## Methods

### Objectives

This systematic review will achieve the following specific objectives:

1. Identify relevant quantitative research on parental childhood VH with vaccine uptake and vaccination intention being relevant outcomes;
2. Identify context-specific causes, behaviour, and impact of VH in the UK; and
3. Map the gaps in knowledge on VH to develop suggestions for further research and to guide interventions in this field.

### Search strategy

To reflect the diverse range of subject areas covered by VH, databases in medicine, nursing, public health, biological and social sciences, behavioural sciences, and psychology were used in this review. The search was also extended to relevant internet sites including Google Scholar and WHO’s Global Literature on Coronavirus Disease. The database search (see Table 1) was supplemented by a manual search of the reference lists of the included studies, as well as the cited references. The search strategy incorporated MeSH or equivalent terms.

**Table 1.** Selected databases

Medical Literature Analysis and Retrieval System Online (MEDLINE)
Cumulative Index of Nursing and Allied Health Literature (CINAHL)
Psychology & Behavioural Sciences Collection
Child Development & Adolescent Studies
Education Research Complete
Google Scholar
WHO’s Global Literature on Coronavirus Disease

Multiple search terms were first developed and then these were combined using the Boolean operators ‘OR’ and ‘AND’. The search for data involved keywords, related terms, variants, or the same meaning for the terminologies (see Table 2).

**Table 2.** Keywords used in search strategy

	OR	OR	AND	OR
vaccination	vaccin*	hesitancy	Parent	child
immunisation	immuniz*	refusal	Caregiver	children
immunisation	immunis*	denial	Guardian	childhood
prevention and control		rejection		
		Anti-vaccination		
		Anti-vax		
		anti-vax		

From the identified search terms, a broad search string was first developed for MEDLINE and then adapted to all other databases. The core search around the concepts of vaccination and hesitancy is shown in Table 3.

**Table 3.** Search string for selected databases

Databases: Medical Literature Analysis and Retrieval System Online (MEDLINE), Cumulative Index of Nursing and Allied Health Literature (CINAHL), Psychology & Behavioural Sciences Collection, Child Development & Adolescent Studies, and Education Research Complete.
Vaccination or immunisation or immunisation or prevention and control Vaccin or immuniz or immunis Hesitancy or refusal or denial or rejection or anti-vaccination or anti-vax 1 or 2 or 3 Parent or caregiver or guardian Child or children or childhood 5 or 6 4 AND 7

The publication dates of interest were limited to the period between 1 January 1998 and 31 December 2020. The starting year was chosen as it was the year of publication of the now-retracted Andrew Wakefield’s article that linked measles, mumps and rubella (MMR) vaccine with the occurrence of autism and behavioural abnormalities in children (Wakefield *et al.*, 1998). The controversy fuelled the anti-vaccination movement (Balakrishnan, 2019; Glasper, 2022). The initial search was conducted from 31 December 2020 to 21 January 2021. The search process and resulting analysis followed the PRISMA (Preferred Reporting Items for Systematic reviews and Meta-analyses) approach (Page *et al.*, 2021).

After the removal of duplicates, the remaining articles were screened by title and abstract. Articles were then excluded using a set of exclusion criteria (Table 4). As this review focused on parental childhood VH with vaccine uptake and vaccination intention being relevant outcomes, articles were excluded for the following reasons: not addressing human vaccines; studies that measured hesitancy indicators on vaccines unrelated to childhood immunisation including adolescent vaccines (Human papillomavirus, Diphtheria-Tetanus-Pertussis booster) and adult vaccines (herpes zoster vaccine); studies not related to determinants of general VH (eg, studies about vaccine efficacy); studies with determinants not linked to a behavioural outcome; modelling studies and intervention studies. Studies without full texts were also excluded. Preprints, grey literature, including dissertations/theses, government publications and articles on mandates were excluded, as these are not peer-reviewed. Other systematic reviews, meta-analysis and review articles were excluded to avoid duplication of studies. Only articles written in English were considered.

**Table 4.** Exclusion criteria

1.	Books or book chapters	12.	Studies not addressing human vaccines
2.	Editorials or letters	13.	Studies not related to parental vaccine hesitancy and healthcare fields of research
3.	Practice guidelines	14.	Studies not related to determinants of parental vaccine hesitancy
4.	Government publications and articles on mandates	15.	Studies that are not peer-reviewed
5.	Papers without abstract	16.	Studies with determinants not linked to a behavioural outcome
6.	Abstract only reports	17.	Studies not reporting primary data (including other reviews and meta-analysis)
7.	Dissertations or theses	18.	Modelling studies
8.	Commentaries	19.	Intervention studies
9.	Preprints	20.	Studies not published in English
10.	Studies without full texts	21.	Studies not published between 1998 and 2020
11.	Studies that measure hesitancy indicators on vaccines that are not related to childhood immunisation including adolescent vaccines (HPV, DTaP booster, etc), seasonal influenza vaccine and adult vaccines (herpes zoster vaccine)	22.	Studies not reporting multivariate analysis of determinants

Filters were provided by most databases for elements of the exclusion criteria, including publication dates (1998–2020), language (English), and type of publication (peer-reviewed journal article). These filters were used during the initial search, when applicable.

### Data extraction

Included studies were coded by publication year, country, WHO region, vaccine, outcome variable (intention or behaviour), and population, among other variables. The predictors of childhood vaccine uptake or intention (*P*-value <0.05) as well as the prevalence rates of parental childhood VH were extracted from the selected studies and documented.

### Quality assessment

To assess the quality of included studies, the Joanna Briggs Institute (JBI) critical appraisal checklist for studies reporting prevalence data was used (Munn *et al.*, 2015) (see Supplementary Material 1). This is a standard, recommended and widely used tool with a higher methodological rigour compared to other appraisal methods (Ma *et al*., 2020; Migliavacaa *et al.*, 2020). The 34 included studies met all the JBI criteria.

### Data synthesis

Thematic analysis was used for the synthesis, analysis, and interpretation of the patterns of meanings, attributes, and findings from the selected quantitative studies (Braun & Clarke, 2006; Guest *et al.*, 2012). A meta-analysis of numerical data was considered inappropriate for this review as the included studies are heterogeneous, clinically diverse, with different metrics or outcomes evaluated, and as such too dissimilar to combine the results (Higgins *et al.*, 2021).

## Results

### Identified literature

**Fig. 3.**
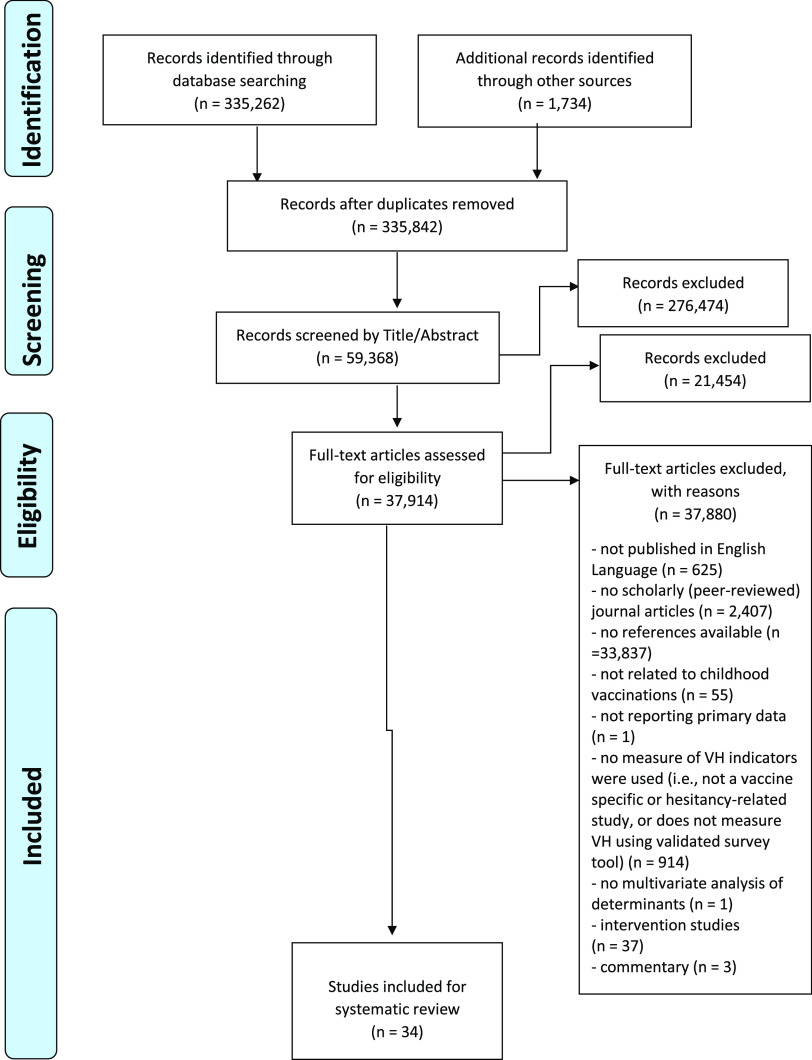
PRISMA flow diagram

In total, 335 262 records (all languages) were identified from the databases using the search strategy previously described (Table 2). An additional 1734 articles were added from other sources (relevant internet sites including Google Scholar and WHO’s Global Literature on Coronavirus Disease, and studies obtained from manual search of the reference lists of the included studies, as well as the cited references). After the removal of duplicates, 335 842 records were shortlisted for screening by title and abstract (Figure 3). A total of 276 474 papers were removed according to the exclusion criteria (Table 4). In all, 37 914 articles were eligible for the full-text assessment. After full-text analysis, 37 880 articles were removed. The remaining articles were considered for descriptive analysis and synthesis (*n* = 34).

A total of 30 articles assessed the prevalence of parental childhood VH in various populations, while four evaluated the intention of parents to vaccinate their children against VPDs (see Supplementary Material 2).

### Descriptive analysis of articles

#### Study setting, design, and sampling

Relevant research about VH was found across all WHO regions. Twelve articles in the current review present findings from the Americas (USA, Canada, Guatemala). From 1998, nine studies came from Europe (Italy, France, UK, Netherlands, Slovenia, Kyrgyzstan), five from Western Pacific (Malaysia, China), two from East Mediterranean (Pakistan, Saudi Arabia), one from South-East Asia (India), one from Africa (Ghana), and four from cross-national comparisons of countries across different regions. Only two studies were conducted in the UK (two further studies included participants from the UK).

The majority of articles present data derived from cross-sectional (72.5%; *n* = 29) study designs. Longitudinal (*n* = 2) and mixed methods (*n* = 3) made up the remaining 15% of study designs used by the selected studies. The articles covered diverse ethnic backgrounds of sample populations. Nearly half (15/34) of the studies examined a multi-ethnic sample. Nineteen studies reported no information on race/ethnicity.

There was an increase in research on parental childhood VH across all WHO regions over the period 1998–2020. There was particular interest shown in pandemic influenza and seasonal influenza vaccines and the newly introduced COVID-19 vaccines. The main outcome variable in most studies (30/34) was actual vaccine behaviour, while the intention to vaccinate against COVID-19 or any new VPD was assessed in 4 of the 34 studies. Childhood vaccines remained a primary focus in Africa, South-Eastern Asia, and East Mediterranean regions. Studies from the Americas, Europe, and Western Pacific considered all age groups, with a tendency to shift to adolescent and adult vaccines. The introduction of COVID-19 vaccines oversaw an increase in published literature on VH with a shift in focus to the adult population. This research boost reflects the extent of the challenges surrounding uptake of the COVID-19 vaccines and the broader implications for vaccine confidence (Bell *et al.*, 2020; Goldman *et al.*, 2020).

#### Focus on specific vaccines

The majority of the selected studies (24/34) considered vaccines in general and were not focused on a specific vaccine. Studies that were specific to one vaccine looked at influenza, MMR, or COVID-19 vaccine, and this was more common in the Americas, Europe, and South-East Asia. Of the 34 articles reviewed, parents or primary caregivers were the focal point, offering their perspectives on the factors influencing their intention to recommend vaccines.

#### Use of theoretical models

Only a few of the studies expanded the field of VH research using novel approaches drawn from the core concepts of social cognitive models. For example, a cross-sectional study in the Netherlands was conducted to determine parental attitudes towards future childhood immunisations (Hak *et al.*, 2005). Guided by the Health Belief Model (HBM), the authors developed a questionnaire for distribution to 800 highly educated parents of children <5 years of age attending day-care centres. With a response rate of 35% and less than half (46%) of participants expressing desire to vaccinate their children against diseases, this study highlighted the need for continuous health education to ensure the success of vaccination programmes. The low predictive capability of HBM variables was evident in this research, given the lack of depth of the questions used to assess and predict behaviour change among the respondents. For example, while questions related to perceived barriers and perceived benefits were strong predictors, questions about perceived severity were weakly correlated.

In Canada, Dubé *et al.* (2018) explored VH among parents and examined factors associated with their intention to vaccinate their children. Informed by the Theory of Planned Behaviour (TPB), this cross-sectional study assessed the relationships between knowledge, attitudes, and beliefs of 2013 parents and their intention to present their children for future vaccinations. Despite the importance of this study, the applied model did not comprehensively cover the influence of broader contextual factors. Even the authors acknowledged that ‘…the predictive power of TPB could be further increased by integrating concepts of risk perception, past behaviour, knowledge, and experience into the model’ (Dubé *et al.*, 2018:547).

A more recent cross-sectional study in China evaluated parental VH and identified risk factors associated with the intention of mothers to vaccinate their children (Hu *et al.*, 2019). Of the 770 mothers of children aged 24–35 months surveyed in Zhejiang province, 79.6% had positive attitudes towards vaccination. Like the Canadian study, this research used the TPB constructs to predict behaviour change among the sample population, and thus had similar shortcomings.

### Analysis of factors (determinants of VH)

The 34 studies recognised the complex nature of VH, evidenced by the range of factors identified as determinants of vaccination behaviour (Table 5). These factors clustered around the core concepts of commonly used social cognitive models such as the HBM, Theory of Reasoned Action, TPB, Social Cognitive Theory, Health Action Process Approach, and the PMT. With respect to the objectives of this review, these findings validate the determinants of VH outlined in the SAGE WG model (MacDonald, 2015). These themes will be adopted for the analysis of factors identified in the selected studies.

**Table 5.** Determinants of vaccine hesitancy

Contextual Influences	Individual and Social Group Influences	Vaccine and Vaccination-Specific Issues
Socioeconomic groups	Experience with past vaccination	Evidence-based risk/benefit analyses
Religion/culture/gender	Perceived risk/benefits	Vaccination schedule
Policies and mandates	Personal experience with and trust in health system and provider	Mode of administration
Influential leaders and individuals	Knowledge/awareness of vaccines	Mode of delivery
Communication and media environment	Beliefs, attitudes and motivation about health and prevention	Introduction of a new vaccine or new formulation
Pharmaceutical industry	Need for vaccines	Reliability of vaccine supply
Historical influences		Role of healthcare professionals
Geographic barriers		Costs
		Tailoring vaccines/vaccination to needs

#### Contextual influences

##### Socioeconomic Factors

Socioeconomic status (SES) was recognised as a significant determinant of VH in nine of the included studies. In Netherlands (Hak *et al.*, 2005), Malaysia (Azizi *et al.*, 2017), France (Bocquier *et al.*, 2018), and China (Hu *et al.*, 2019), low SES was identified as a promoter/enabler of vaccination, while high SES was found to be a barrier. Another study in Malaysia (Kalok *et al.*, 2020) indicated low SES as a barrier to vaccination, whereas high SES was considered a promoter. This finding was corroborated by studies in India (Dasgupta *et al.*, 2018) and Pakistan (Khattak *et al.*, 2021). In China, although caregivers with high SES accepted vaccines with doubts, they did not delay or refuse vaccines for their children (Fanxing *et al.*, 2020). The varying results obtained by different studies reflect the multidimensional nature of VH, especially in the context of socioeconomic and health disparities existing among nations (Bocquier *et al.*, 2018). Thus, it would be counterproductive to consider individual factors in isolation as multiple influences are at play (Larson *et al.*, 2015a; Larson *et al.*, 2015b).

##### Communication and media environment

The mass media remains a regular source of information about vaccination and vaccine-related issues. Constant exposure to vaccination stories could serve as a promoter of, or barrier to, vaccination (Larson *et al.*, 2015b). Previous studies in Nigeria (Antai, 2009; Babalola & Lawan, 2009; Babalola, 2011), India (Patra, 2012), and Bangladesh (Rahman & Obaida-Nasrin, 2010) highlighted the positive association between the media and the promotion of vaccination. In this review, however, negative news stories acted as a barrier to vaccination, as seen in studies from the UK (Campbell *et al.*, 2017), Canada (Greenberg *et al.*, 2017; Dubé *et al.*, 2018), France (Bocquier *et al.*, 2018), Italy (Napolitano *et al.*, 2018; Bianco *et al.*, 2019), Slovenia (Ucakar *et al.*, 2018), Malaysia (Azizi *et al.*, 2017; Musa *et al.*, 2019; Kalok *et al.*, 2020), Pakistan (Khattak *et al.*, 2021), Saudi Arabia (Alsubaie *et al.*, 2019), and China (Hu *et al.*, 2019; Fanxing *et al.*, 2020).

##### Trust in pharmaceutical companies

In nine of the included studies, parents had a mistrust of pharmaceutical industries, believing that economic interests influenced vaccination policy (Gilkey *et al.*, 2016; Greenberg *et al.*, 2017; Bocquier *et al.*, 2018; Domek *et al.*, 2018; Dubé *et al.*, 2018; Giambi *et al.*, 2018; Bianco *et al.*, 2019; Alsubaie *et al.*, 2019; Musa *et al.*, 2019). Parents felt the pharmaceutical sector should act in the public’s best interest. Lack of trust in pharmaceutical companies was thus considered a barrier to vaccination.

Parents questioning the intentions of pharmaceutical companies may result in weak public acceptance of vaccines (Alsubaie *et al.*, 2019). Therefore, it is important to consider how parents view the pharmaceutical sector as a major factor in the mistrust that leads to vaccine refusal.

#### Individual and social group influences

##### Past experiences

Negative past experiences with vaccination services, such as side effects, poor continuity of care or lack of compassionate or comprehensive care, were significant predictors of VH among parents in twelve of the studies reviewed. These studies were split across the USA (Henrikson *et al.*, 2017), Canada (Dubé *et al.*, 2018), Italy (Giambi *et al.*, 2018, Napolitano *et al.*, 2018), Bianco *et al.*, 2019), China (Hu *et al.*, 2019; Fanxing *et al.*, 2020), Malaysia (Musa *et al.*, 2019), and Ghana (Wallace *et al.*, 2019). Three multinational studies also reported these findings (Bakhache *et al.*, 2013; Larson *et al.*, 2015b; Goldman *et al.*, 2020).

##### Beliefs and attitudes

The importance of beliefs about vaccine safety and efficacy, and general attitudes and trust were noted by all 34 studies reviewed. These factors were significantly associated with the vaccination status of children. Having a positive attitude towards vaccination and a belief in the scientific efficacy of vaccines were identified as promoters of vaccination (Opel *et al.*, 2011b; Opel *et al.*, 2013; Strelitz *et al*., 2015; Azizi *et al.*, 2017; Campbell *et al.*, 2017; Henrikson *et al.*, 2017; Bocquier *et al.*, 2018; Dubé *et al.*, 2018; Napolitano *et al.*, 2018; Rachel *et al.*, 2018; Bianco *et al.*, 2019; Dubé *et al.*, 2019; Musa *et al.*, 2019; Kalok *et al.*, 2020). On the other hand, anti-vaccination behaviours, preference for alternative health approaches, and a belief in myths, rumours, or conspiracy theories acted as barriers to vaccination (Larson *et al.*, 2015b; Azizi *et al.*, 2017; Greenberg *et al.*, 2017; Bocquier *et al.*, 2018; Dubé *et al.*, 2018; Napolitano *et al.*, 2018; Ucakar *et al.*, 2018; Alsubaie *et al.*, 2019; Bianco *et al.*, 2019; Hu *et al.*, 2019; Musa *et al.*, 2019; Fanxing *et al.*, 2020; Kalok *et al.*, 2020; Khattak *et al.*, 2021).

##### Knowledge and awareness

Knowledge about the severity of a disease and awareness of disease susceptibility were important determinants of the vaccination status of children in the UK (Campbell *et al.*, 2017; Bell *et al.*, 2020), USA (Opel 2011b; Strelitz *et al.,* 2015; Henrikson *et al.*, 2017), Canada (Greenberg *et al.*, 2017; Dubé *et al.*, 2019), Italy (Napolitano *et al.*, 2018), Saudi Arabia (Alsubaie *et al.*, 2019), Malaysia (Azizi *et al.*, 2017; Musa *et al.*, 2019; Kalok *et al.*, 2020), and Ghana (Wallace *et al.*, 2019).

##### Risk-benefit perception (perceived threat vs perceived rewards)

Several studies (22/34) highlighted the influence that perceived risks and benefits have on vaccination behaviour. Eight of these studies came from the Americas (Opel *et al.*, 2011b; Frew *et al.*, 2016; Gilkey *et al.*, 2016; Greenberg *et al.*, 2017; Rachel *et al.*, 2018; Domek *et al.*, 2018; Dubé *et al.*, 2018; Dubé *et al.*, 2019), six from Europe (Hak *et al.*, 2005; Akmatov *et al.*, 2009; Bocquier *et al.*, 2018; Napolitano *et al.*, 2018; Bianco *et al.*, 2019; Bell *et al.*, 2020), four from Western Pacific (Azizi *et al.*, 2017; Hu *et al.*, 2019; Musa *et al.*, 2019; Fanxing *et al.*, 2020), two from East Mediterranean (Alsubaie *et al.*, 2019; Khattak *et al.*, 2021), one from Southeast Asia (Dasgupta *et al.*, 2018), and one from Africa (Wallace *et al.*, 2019). Parents who intend to have their children vaccinated had a lower perceived risk of vaccination and vice versa. If parents perceive the risk of a VPD to be lower than the risk from vaccines, they are likely to doubt the relevance of the vaccines and become vaccine-hesitant. These determinants are in line with the Threat Appraisal Pathway of the PMT: Perceived Threat (Severity, Vulnerability), and Perceived Rewards (Intrinsic Rewards, Extrinsic Rewards).

##### Vaccination as a social norm

Vaccine uptake was influenced by the presence of peers or relatives that are in support of vaccination, as reflected in studies carried out in the USA (Rachel *et al.*, 2018; Henrikson *et al.*, 2017), Canada (Dubé *et al.*, 2018), Italy (Bianco *et al.*, 2019), Netherlands (Hak *et al.*, 2005), Malaysia (Musa *et al.*, 2019; Kalok *et al.*, 2020), and Ghana (Wallace *et al.*, 2019). These studies found that parents who view immunisation as a social responsibility and consider the importance of herd immunity are less likely to be vaccine-hesitant. The need to protect others from harm is a behavioural outcome reflected in the Coping Appraisal Pathway of the PMT.

#### Vaccine and vaccination-specific issues

##### Accessibility

Time, distance, and cost (including cost of transport to a vaccine provider and the cost of self-paid vaccines) were identified as barriers to vaccination in five of the studies reviewed (Larson *et al.*, 2015b; Dasgupta *et al.*, 2018; Domek *et al.*, 2018; Musa *et al.*, 2019; Fanxing *et al.*, 2020). In Guatemala (Domek *et al.*, 2018), perceived cost (another component of the Coping Appraisal Pathway of the PMT) was more important in urban areas than rural areas. Only in Pakistan (Khattak *et al.*, 2021) did time, distance to clinic and cost not deter parents from accessing vaccination services.

##### Introduction to a new vaccine

Parental concern about new vaccines carrying more risk than older vaccines had a negative association with the intention to vaccinate in the USA (Allred *et al.*, 2005), Netherlands (Hak *et al.*, 2005), Italy (Giambi *et al.*, 2018), Guatemala (Domek *et al.*, 2018), Malaysia (Musa *et al.*, 2019), India (Dasgupta *et al.*, 2018), Saudi Arabia (Alsubaie *et al.*, 2019), and Pakistan (Khattak *et al.*, 2021). However, a multinational survey of seven countries (UK, Canada, Australia, France, Spain, Germany, and Sweden) reported that parents would welcome the introduction of new vaccines, even if it requires additional clinic visits or coadministration with current vaccines (Bakhache *et al.*, 2013).

##### Role of healthcare professionals

All 34 studies acknowledged that advice or recommendation from health professionals could be an important determinant of vaccine acceptance. Parents who do not trust healthcare personnel or have little faith in the health system are more likely to be vaccine-hesitant. The studies suggest the need for healthcare providers to use their privileged position to address parental concerns about vaccinations, as this could influence the decision-making process.

## Discussion

For the period under review (1998–2020), relevant studies about VH were found across all WHO regions, with the majority from the Americas and Europe. This does not necessarily suggest an increased prevalence of VH and issues related to vaccine acceptance in these regions, as focus may not be on vaccination, but on treatment of VPDs (eg, influenza, measles, mumps, varicella, pertussis, and meningococcal disease). However, as most of the world’s population lives in other regions, it is difficult to make inferences about the scarcity of available research in those parts.

Several determinants of VH were identified by the studies included in this review. No single algorithm was applicable to all studies as each factor was independent and varied across time, place, and vaccines, reflecting the complex interplay of other variables and the context-specific nature of VH (Clark & Sanderson, 2009; Larson *et al.*, 2015a, 2015b). Even in parts of the world where research was readily available, only few studies examined the different levels of interactions that exist between factors influencing VH. Different research methods were applied, and most of the studies were cross-sectional, thus making it difficult to draw conclusions about the influence of single or multiple determinants of vaccine acceptance at the individual or collective level. Future research should consider qualitative studies to help fill these gaps and contribute to existing knowledge and understanding of the many factors that influence parental decision-making.

The quantitative studies considered in this review examined the determinants of vaccine acceptance such as lack of vaccination awareness, fear of side effects, mistrust in the healthcare system and health professionals, poor perception of vaccine value, and negative past experiences with vaccine services, among others. However, it is difficult to make inferences about the relative strength of influence of these determinants because the studies were rarely based on theoretical models. While these findings do not rule out the significance of identified factors, they do highlight the shortcomings in such approaches.

Most studies showed that sociodemographic factors are important drivers of VH. It is crucial to emphasise, however, that most sociodemographic factors play a minor role in explaining individual VH. In the sociodemographic variables section, for example, inconsistent results were commonly reported. Furthermore, sociodemographic characteristics are at best a collection of plausible causes and can never fully define a particular behaviour without additional analysis (Schmid *et al.*, 2017). Several studies, for example, suggested a link between a study population’s race/ethnicity, sex distribution, and vaccination intention (Allred *et al.*, 2005; Strelitz *et al.,* 2015; Gilkey *et al.*, 2016; Musa *et al.*, 2019; Bell *et al.*, 2020; Kalok *et al.*, 2020; Khattak *et al.*, 2021). These associations could be accounted for by other factors such as family size (Luyten *et al*., 2019), access to healthcare facilities (Lockyer *et al.*, 2021), healthcare provider discrimination (Woolf *et al.*, 2021), misinformation on social media (Broadbent, 2019), trust in government and/or health authorities (Trent *et al.*, 2022), attitudes towards vaccination (Gravelle *et al.*, 2022), and the fear of vaccine side effects (Karafillakis *et al.*, 2016). As a result, sociodemographic variables such as ethnicity, race, and gender are only carrier variables, not explanatory variables (Schmid *et al.*, 2017). This suggests that these variables could be confounders of the variables that actually cause VH. While such factors may be associated with VH, they cannot explain the development or severity of the situation. Most significantly, they are unhelpful in informing decisions to overcome hesitancy if psychological determinants are ignored. While these carrier variables may be useful in identifying target groups for intervention programmes, they should not be used to design the intervention (Schmid *et al.*, 2017).

Across the target demographics, all the explanations for not being vaccinated as stated by the 5C model were recognised as major barriers to vaccine acceptance. Constraints and calculation, however, were less significant drivers. For pandemic influenza, the most common reasons for apprehension were a loss of faith in authorities and a diminished perception of the vaccine’s safety, as well as complacency, largely caused by low perceived risk and fear about the infection. The most common causes of VH for seasonal influenza vaccination were a lack of faith in authority, low vaccine effectiveness, low vaccine safety perceptions, vaccine misconceptions, and a negative attitude toward vaccines. A loss of confidence due to low perceived vaccination efficacy was commonly noted for both flu strains. COVID-19 immunisation intention was most significantly linked to confidence and collective responsibility.

The benefits of using the 5C model to design interventions can be seen in the distinctions between disease types in terms of their psychological profile of vaccine denial in target populations. The model serves as a framework for identifying, developing, and implementing effective solutions to the VH crisis (Betsch *et al.*, 2015). If one wants to enhance COVID-19 vaccine uptake in the hospital environment, for example, the findings of this review show that tackling confidence issues (by dispelling myths and making people understand the ethical and professional need to get vaccinated) is a viable mechanism. Low confidence has been demonstrated to respond well to informational interventions such as instructional initiatives (Betsch *et al.*, 2015). It has also been demonstrated that structural interventions such as compulsory vaccinations, which are effective in overcoming complacency, should be approached with caution, as negative attitudes regarding immunisation are substantial obstacles that can lead to reactance after structural intervention efforts (Betsch *et al.*, 2015). When the findings of this systematic review are integrated with conceptual frameworks such as the 5C model, important revelations about modifiable behaviours can emerge.

Campaigns aimed at raising parental vaccination intention would most likely be effective if they emphasise building confidence and collective responsibility while reducing complacency. Other factors, such as constraints and calculation, had smaller negative correlations with vaccination intention. When developing solutions, the psychological characteristics that underpin these motivations should be considered. Vaccination intention is influenced by variations in levels of confidence, which are driven by the perceived risk and safety profiles of vaccines. Because parents who believe vaccines have greater risks than benefits have lower levels of confidence, the importance of faith in the government and health officials in clarifying vaccine intentions is vital. Parents that have less faith in these institutions have lower confidence levels, which leads to a lower intention to get vaccinated. Vaccination intention is also influenced by the extent to which family members and friends express their need to get vaccinated.

Complacency sets in when the perceived dangers of VPDs are low, and vaccination is not considered an essential preventive measure (MacDonald, 2015). Individuals who are unconcerned about communicable diseases do not feel threatened by them and hence do not feel compelled to change their preventative habits (Schwarzer & Fuchs, 1996). Because of the low level of involvement, the affected people do not see the need to actively seek information and increase their knowledge and awareness of prevailing issues (Fischer *et al.*, 2011). Preventive behaviour is also not perceived as a descriptive or injunctive norm in society; therefore, it is regarded as separate from subjective norms (Askelson *et al.*, 2010). However, complacency should be linked to a poor perception of disease risks (Brewer *et al.*, 2007).

Because prevention is a future-oriented behaviour, it is expected to have a negative relationship with the consideration of future repercussions (Petrocelli, 2003). Individuals with a high level of complacency should also have a favourable risk perception, showing a propensity for risk-taking behaviours, because future repercussions are irrelevant (Johnson *et al.*, 2004). This may be linked to perceptions of invulnerability as well as a positive subjective personal health status (Lapsley & Hill, 2010).

Parents who believe the risk of VPDs in their surroundings is minimal have a decreased intention to vaccinate their children, owing to a reduced desire to safeguard others. Furthermore, personality plays a key role in understanding how vaccination is viewed as a social responsibility. Psychopathic qualities, which are linked to antisocial behaviour caused by a lack of empathy, emotion, and self-control (Jones & Paulhus, 2014), have a negative relationship with collective responsibility and, as a result, with vaccination intentions. Likewise, parents with more humane characteristics, such as those who feel greater sympathy for others and wish to help those in need, have a stronger intention to vaccinate their children because they have a larger sense of community duty.

Research suggests that attempting to boost both confidence and collective responsibility at the same time will be beneficial, as interventions that target multiple underlying factors have proved to be more effective (Frew & Lutz, 2017). The results of this study suggest that is critical to target vaccine safety and efficacy when addressing confidence. Concerns regarding safety, vaccine side effects, speed of development, and the desire for the vaccine to be shown efficient and safe over a longer period were the most common reasons given in this review for COVID-19 VH. Confidence levels in the vaccine can be boosted by debunking myths about the vaccine and offering real information on issues such as why the vaccine was produced so quickly, for example. Nevertheless, it is critical to consider the way this information is communicated, and the personnel involved, because a correction of information could backfire and lead to even more polarised sentiments among those who already have strong opinions (Glaeser & Sunstein, 2014). Because in this study, poor confidence was linked to a distrust of government and healthcare agencies, safety, and efficacy information should best be presented by people who are not in typical positions of authority. A viable approach would be to use people who are considered as reputable by the target audience but are not expected to give this knowledge (Glaeser & Sunstein, 2014). Campaigns involving peers or celebrities, for example, could be used to reach parents.

In this study, parents’ collective responsibility was shown to significantly predict COVID-19 vaccine uptake. The potential threat of COVID-19 for other family members in a household environment indirectly influences parental vaccination intention. The presence of family members who are susceptible to COVID-19, such as those with underlying medical conditions, could motivate parents to get their children vaccinated, thus safeguarding the people around them. Vaccination programmes focused on parents may thus be more effective if they highlight the hazards to individuals in the immediate vicinity of the parents. Vaccination is an effective way to explain what herd immunity is about (Betsch *et al.*, 2017). When deciding whether to vaccinate their children, parents can and should be made aware that they are making a collective decision, not simply an individual one. To raise awareness, campaigns could address the reasons why certain people cannot get vaccinated (eg, those who have had an adverse reaction to immunisations, have autoimmune diseases, or have other illnesses).

Because parents with less altruistic, assertive, and gregarious personalities are less likely to feel communal responsibility, it will be difficult, if not impossible, to influence these personality traits. However, because these parents have less empathy for others, campaigns emphasising the vaccine’s prosocial effects may not be enough to sway certain groups and may even compound the free-rider problem (Ibuka *et al.*, 2014). As a result, it is critical to keep expressing the personal hazards of COVID-19 to parents, such as the possibility of long-term negative effects of COVID-19 (Mahase, 2020).

Descriptive norms can influence vaccination intention indirectly through confidence and complacency, just as they can influence the decision-making process directly. These norms have been shown to be powerful motivators of behaviour, particularly in uncertain times (Cialdini, 2009). Vaccination campaigns may be more effective if they emphasise the importance of vaccination among parents by emphasising that most families plan to get vaccinated.

When family members have already been vaccinated, the level of collective responsibility may be reduced due to a lower perceived risk of VPDs for others. As a result, it is critical that parent-focused efforts begin early on, when the importance of vaccination is most apparent, and thus, positive attitudes can be formed. According to studies, once a sufficient decision has been made to get vaccinated, it is more likely to be followed through (Auslander *et al.*, 2019). In terms of policy, the process of getting vaccinated should be simple, quick, and free of avoidable constraints to accelerate the shift from intention to behaviour (DaCosta DiBonaventura & Chapman, 2005).

## Limitations, directions for future research, and conclusions

### Limitations

Rather than obtaining a comparison of the individual determinants of vaccine acceptance, this systematic review analysed the spectrum of parental childhood VH and its drivers. Studies that investigated the different barriers but found no significant connections are not reported or considered since they were outside the scope of the research. A meta-analytic technique is required to assess the cumulative outcome measures of relevant barriers and their respective significance. However, meta-analytic approaches to addressing VH have significant challenges because the outcome measures are not frequently based on the constructs of theoretical models and their use varies widely among researchers.

Most of the VH studies were undertaken in the United States and Europe. All other jurisdictions were relatively poorly represented. Even though research for the target populations has increased in number over time, the number of studies focusing on children has remained comparatively low. As a result of the scarcity of data, the results of this review must be confined to the locations and populations that are accessible.

The review had other limitations, including the exclusion of databases that had articles not written in English, which may have affected the sensitivity of searches in other languages, and the exclusion of government publications and articles on mandates, which may have influenced findings around the impact of health policies and practices.

Notwithstanding the shortcomings, this research offers governments and public health experts the necessary tools for understanding the key drivers of vaccination behaviour and vaccination intention among parents. Considering the fluctuating rates of vaccine acceptance in the studies reviewed, it is hoped that the findings of this study will aid in the development and enhancement of public health interventions to improve vaccine compliance above the proportions required for herd immunity.

### Directions for future research

Underserved regions where only limited studies were found on parental childhood VH and demographics (eg, parents of children aged 0–6 years) should be the focus of future studies. From the results of this research, the UK is one of those regions that requires further investigations. More research will provide further evidence to design interventions across the UK and all WHO regions and for all groups at risk of VPDs.

Studies should not only concentrate on regions and demographics but also on measurable outcomes. Psychological variables can help researchers further comprehend why some people reject vaccinations while others do not. These variables are not studied regularly. Psychological principles are rarely employed in the measurement of study outcomes, and the tools used to evaluate the constructs differ significantly between investigations. Furthermore, risk perception variables are hardly distinguished and used interchangeably throughout and even within articles.

Theory-based psychological scales should be adopted for use in research to obtain accurate results and allow the scientific community to compare findings across publications. This approach will ensure scientific advancement in the relatively new field of VH research and raise the standard of future investigations.

### Conclusions

The emergence of VH has been central to the understanding of the wider concept of vaccine acceptance. This review showed that unfavourable dispositions towards vaccinations and behavioural attitudes such as a reduced perception of vaccine effectiveness and mistrust of health authorities were the most often cited barriers to vaccine uptake. Other evaluations include concerns about vaccine safety, low perceived severity of VPDs, and low perceived disease susceptibility.

Confidence and complacency, according to available evidence, are major determinants of VH. Anxiety, low perceived risk, and low disease severity were the most common signs of complacency. Doubts about vaccine safety and effectiveness, as well as lack of faith in health officials and the assumption that vaccines can cause the diseases they were meant to prevent, all contributed to a lack of confidence.

The constructs of relevant theoretical models have provided further context to the evolution of VH determinants, emphasising the need for parents and stakeholders to be actively engaged in the decision-making process from an early stage. It is clear, however, that additional information sources are needed to ensure these models adequately account for the influence of broader contextual factors, particularly in regions with limited peer-reviewed literature.

Theoretical approaches to quantifying VH will continue to strengthen the body of knowledge needed to develop successful evidence-based interventions. The efficacy of vaccine advocacy campaigns could be increased and the burden of VPDs could be lowered by adopting clinical, patient-centred techniques to measure and overcome VH. A combination of local, regional, and universally driven initiatives will be critical in the early detection of parental concerns.

## Supplementary material

For supplementary material accompanying this paper visit http://doi.org/10.1017/S1463423622000512. click here to view supplementary material
