# Supplementary material for: Parental childhood vaccine hesitancy and predicting uptake of vaccinations: a systematic review
Source: Prim Health Care Res Dev. 2022 Nov 4;23:e68. doi: 10.1017/S1463423622000512 (PMC9641700; doi:10.1017/S1463423622000512)
Supplement: Supplementary file 1 [file phcsup.zip › S1463423622000512sup001.docx]

Supplementary Material 1: Quality Assessment

| Study | Checklist | | | | | | | | | Overall Appraisal |
| --- | --- | --- | --- | --- | --- | --- | --- | --- | --- | --- |
|  | Was the sample frame appropriate to address the target population? | Were study participants sampled in an appropriate way? | Was the sample size adequate? | Were the study subjects and the setting described in detail? | Was the data analysis conducted with sufficient coverage of the identified sample? | Were valid methods used for the identification of the condition (VH or Intention to vaccinate)? | Was the condition measured in a standard, reliable way for all participants? | Was there appropriate statistical analysis? | Was the response rate adequate, and if not, was the low response rate managed appropriately? |  |
| Akmatov et al., 2009 | Yes | Yes | Yes | Yes | Yes | No | No | Yes | Yes | Inclue |
| Alsubaie et al., 2019 | Yes | Yes | Yes | Yes | Yes | Yes | Yes | Yes | Yes | Include |
| Allred et al., 2005 | Yes | Yes | Yes | Yes | Yes | No | No | Yes | Yes | Include |
| Azizi et al., 2017 | Yes | Yes | Yes | Yes | Yes | Yes | Yes | Yes | Yes | Include |
| Bakhache et al., 2013 | Yes | Yes | Yes | Yes | Yes | No | No | Yes | Yes | Include |
| Bell et al., 2020 | Yes | Yes | Yes | Yes | Yes | No | No | Yes | Yes | Include |
| Bianco et al., 2019 | Yes | Yes | Yes | Yes | Yes | Yes | Yes | Yes | Yes | Include |
| Bocquier et al., 2018 | Yes | Yes | Yes | Yes | Yes | Yes | Yes | Yes | Yes | Include |
| Campbell et al., 2017 | Yes | Yes | Yes | Yes | No | No | No | No | Yes | Include |
| Dasgupta et al., 2018 | Yes | Yes | Yes | Yes | Yes | Yes | Yes | Yes | Yes | Include |
| Domek et al., 2018 | Yes | Yes | Yes | Yes | Yes | Yes | Yes | Yes | Yes | Include |
| Dube et al., 2018 | Yes | Yes | Yes | Yes | Yes | Yes | Yes | Yes | Yes | Include |
| Dube et al., 2019 | Yes | Yes | Yes | Yes | Yes | Yes | Yes | Yes | Yes | Include |
| Fanxing et al., 2020 | Yes | Yes | Yes | Yes | Yes | Yes | Yes | Yes | Yes | Include |
| Frew et al., 2016 | Yes | Yes | Yes | Yes | Yes | No | No | Yes | Yes | Include |
| Giambi et al., 2018 | Yes | Yes | Yes | Yes | Yes | Yes | Yes | Yes | Yes | Include |
| Gilkey et al., 2016 | Yes | Yes | Yes | Yes | Yes | Yes | Yes | Yes | Yes | Include |
| Goldman et al., 2020 | Yes | Yes | Yes | Yes | Yes | Yes | Yes | Yes | Yes | Include |
| Greenberg et al., 2017 | Yes | Yes | Yes | Yes | Yes | No | No | Yes | Yes | Include |
| Larson et al., 2015b | Yes | Yes | Yes | Yes | Yes | Yes | Yes | Yes | Yes | Include |
| Hak et al., 2005 | Yes | Yes | Yes | Yes | Yes | Yes | Yes | Yes | Yes | Include |
| Henrikson et al, 2017 | Yes | Yes | Yes | Yes | Yes | Yes | Yes | Yes | Yes | Include |
| Hu et al., 2019 | Yes | No | Yes | Yes | Yes | No | No | Yes | Yes | Include |
| Kalok et al., 2020 | Yes | Yes | Yes | Yes | Yes | Yes | Yes | Yes | Yes | Include |
| Khattak et al., 2020 | Yes | Yes | Yes | Yes | Yes | Yes | Yes | Yes | Yes | Include |
| Musa et al., 2019 | Yes | Yes | Yes | Yes | Yes | Yes | Yes | Yes | Yes | Include |
| Napolitano et al., 2018 | Yes | Yes | Yes | Yes | Yes | Yes | Yes | Yes | Yes | Include |
| Opel et al., 2011b | Yes | Yes | Yes | Yes | Yes | Yes | Yes | Yes | Yes | Include |
| Opel et al., 2013 | Yes | Yes | Yes | Yes | Yes | Yes | Yes | Yes | Yes | Include |
| Rachel et al., 2017 | Yes | Yes | Yes | Yes | Yes | Yes | Yes | Yes | Yes | Include |
| Stefanoff et al., 2010 | Yes | Yes | Yes | Yes | Yes | Yes | Yes | Yes | Yes | Include |
| Strelitz et al., 2015 | Yes | Yes | Yes | Yes | Yes | Yes | Yes | Yes | Yes | Include |
| Ucakar et al., 2018 | Yes | Yes | Yes | Yes | Yes | Yes | Yes | Yes | Yes | Include |
| Wallace et al., 2019 | Yes | Yes | Yes | Yes | Yes | Yes | Yes | Yes | Yes | Include |
