# Supplementary material for: Parental childhood vaccine hesitancy and predicting uptake of vaccinations: a systematic review
Source: Prim Health Care Res Dev. 2022 Nov 4;23:e68. doi: 10.1017/S1463423622000512 (PMC9641700; doi:10.1017/S1463423622000512)
Supplement: Supplementary file 1 [file phcsup.zip › S1463423622000512sup002.docx]

**Supplementary Material 2: Data Extraction Table**

|  | **Study Details** | **Research Parameters** | **Population**  **And Sample Selection** | **Outcomes, Analytical Methods, Results** | **Notes** |
| --- | --- | --- | --- | --- | --- |
| 1. | **Researcher(s):**  Akmatov et al., 2009  **Title:**  Attitudes and beliefs of parents about childhood vaccinations in post-Soviet countries: The example of Kyrgyzstan  **Year:**  2009  **Journal:**  The Paediatric Infectious Disease Journal  **Volume:**  28  **Country:**  Kyrgyzstan  **WHO Region:**  EUR  **Quality Assessment:**  Include | **What was/were the research question(s):**  What are the attitudes of parents’ regarding childhood vaccinations?  **What theoretical approach (e.g., Grounded Theory) does the study take (if specified):**  Self-administered questionnaire developed for the purpose of the study | **What population were the sample recruited from:**  Parents of first-year school children  **How were they recruited:**  Cluster sampling  **How many participants were recruited:**  934  **Were there specific inclusion criteria:**  Participants from all 4 districts of Bishkek, the capital of Kyrgyzstan.  **Were there specific exclusion criteria:**  None reported (NR)  **Were there specific vaccines under consideration:**  NR  **Other details:**  Most respondents had some form of education (secondary, 24%; higher education, 41%). | **Brief description of method and analytical process:**  Cross-sectional study was conducted. The questions used to assess attitudes toward vaccination were analysed using factor analysis. Categorical Principal Components Analysis was also done, as well as Chi-square test for bivariate analysis.  **Key outcomes relevant to this review**:  Prevalence rates of parental childhood vaccine hesitancy:  3-15% of parents expressed concerns about vaccinations.  Predictors of childhood vaccine uptake or intention (p-value < .25):  Both parents' education levels were linked to low vaccine safety beliefs. Parents whose children had allergies, as well as non-religious respondents and Christians, were more likely to have low safety beliefs.  **How the outcomes were measured:**  In two separate regression models, factors associated with having a low score on vaccine safety beliefs and a high score on antivaccine attitudes were measured. For bivariate analysis, the chi-square test was used, and all variables associated with the outcome variables at P-value < 0.25 were included in a multivariable logistic regression model.  **Potential confounders:**  NR  **Ethical issues:**  NR | **Limitations identified by author(s):**  NR  **Evidence gaps and/or recommendations for future research:**  Future research should evaluate the various dimensions of vaccine-related attitudes, such as safety concerns resulting from information about potential risks or a lack of information about general safety.  **Source of funding:**  NR |
| 2. | **Researcher(s):**  Allred et al., 2005  **Title:**  Parental Vaccine Safety Concerns: Results from the National Immunization Survey, 2001–2002  **Year:**  2005  **Journal:**  American Journal of Preventive Medicine  **Volume:**  28  **Country:**  USA  **WHO Region:**  AMR  **Quality Assessment:**  Include | **What was/were the research question(s):**  What is the association between parental safety beliefs and children’s vaccination status?  **What theoretical approach (e.g., Grounded Theory) does the study take (if specified):**  The Parental Knowledge and Experiences module was adopted for the study. | **What population were the sample recruited from:**  Parents of children aged 19 to 35 months who responded to the National Immunization Survey (NIS) conducted by the Centres for Disease Control and Prevention (CDC)  **How were they recruited:**  Random sapling  **How many participants were recruited:**  7810  **Were there specific inclusion criteria:**  Parents who responded to the NIS  **Were there specific exclusion criteria:**  NR  **Were there specific vaccines under consideration:**  Diphtheria and tetanus toxoids and pertussis vaccine, poliovirus vaccine, MMR vaccine, *Haemophilus influenzae* type b vaccine, and hepatitis B vaccine  **Other details:**  Ethnically diverse sample: Hispanic, 23.8%; Non-Hispanic white, 56.6%; Non-Hispanic black, 14.5%; Non-Hispanic other, 5.1%. | **Brief description of method and analytical process:**  Research was cross-sectional in nature. All analyses were conducted using SAS, release 8.02 (SAS Institute, Cary NC), and SAS-callable SUDAAN, release 8.0.0 (Research Triangle Institute, Research Triangle Park NC).  **Key outcomes relevant to this review**:  Prevalence rates of parental childhood vaccine hesitancy:  6% of parents rated vaccines as neither safe nor unsafe, and 1% as unsafe.  Predictors of childhood vaccine uptake or intention (p-value < .05):  Parental safety belief was significantly associated with a child’s up-to-date vaccination status. The only significant demographic predictor was the child's race/ethnicity. Non-Hispanic whites made up 70% of children whose parents expressed the highest vaccine safety concerns, compared to 56% of children whose parents expressed less concerns.  **How the outcomes were measured:**  The Parental Knowledge and Experiences module was used as the measuring tool. Multivariate logistic regression analyses examined associations between attitudes and up-to-date vaccination coverage.  **Potential confounders:**  NR  **Ethical issues:**  NR | **Limitations identified by author(s):**  Parental attitudes were examined after the bulk of vaccines had been provided at the time of interview; various factors could have impacted their immunisation attitudes over time. Also, other factors affecting vaccine uptake, such as provider practises and system barriers, were not considered.  **Evidence gaps and/or recommendations for future research:**  More research is needed to understand how vaccine safety concerns among parents and providers interact to influence vaccine coverage. Studies should be conducted to determine why there are racial and ethnic differences in vaccine safety.  **Source of funding:**  NR |
| 3. | **Researcher(s):**  Alsubaie et al., 2019  **Title:**  Vaccine hesitancy among Saudi parents and its determinants: Result from the WHO SAGE working group on vaccine hesitancy survey tool  **Year:**  2019  **Journal:**  Saudi Medical Journal  **Volume:**  40  **Country:**  Saudi Arabia  **WHO Region:**  EMR  **Quality Assessment:**  Include | **What was/were the research question(s):**  1) What is the prevalence of vaccine hesitancy and its determinants among Saudi parents?  2) What is the relationship between vaccine hesitancy and a child’s immunization status?  **What theoretical approach (e.g., Grounded Theory) does the study take (if specified):**  The 11-item vaccine hesitancy scale (VHS), designed by the WHO SAGE working group, was adopted for the study. | **What population were the sample recruited from:**  Parents of children aged 2 months to 7 years who were visiting outpatient clinics at King Khalid University Hospital, Riyadh, Kingdom of Saudi Arabia, between July 2017 and October 2018.  **How were they recruited:**  Purposive sampling  **How many participants were recruited:**  500  **Were there specific inclusion criteria:**  Participants visiting outpatient clinics at King Khalid University Hospital, Riyadh, Kingdom of Saudi Arabia, between July 2017 and October 2018.  **Were there specific exclusion criteria:**  Non-Saudi parents and parents of children with an immunodeficiency  **Were there specific vaccines under consideration:**  NR  **Other details:**  Most parents surveyed (90.8%) were female and had a bachelor's degree or higher (60.4%) | **Brief description of method and analytical process:**  Cross-sectional study was conducted. Statistical analysis was done using SPSS version 21 (IBM Corp., Armonk, NY, USA).  **Key outcomes relevant to this review**:  Prevalence rates of parental childhood vaccine hesitancy:  20% of the parents were reluctant or hesitant to get their child vaccinated.  Predictors of childhood vaccine uptake or intention (p-value < .05):  Male gender and parents with a postgraduate degree, such as a master's or PhD, were more vaccine hesitant than parents with a bachelor's or school degree (p<0.001). Furthermore, when compared to parents with older children, parents with children aged 4 years or younger were more likely to report partial vaccination status (p<0.013).  **How the outcomes were measured:**  VHS was the measuring tool. Multivariate logistic regression was used to investigate which variables predicted parents' vaccine hesitancy and partial immunisation status of their child, while controlling for the parents' sociodemographic characteristics (age, gender, education level, number of children in their care, as well as age of youngest child in care).  **Potential confounders:**  NR  **Ethical issues:**  Research was approved by the Institutional Review Board of College of Medicine, King Saud University in Riyadh, KSA. | **Limitations identified by author(s):**  Because KSA lacks a central immunisation registry and the child's vaccination status was reported by parents, this could be underestimated or overestimated due to social desirability or recall bias.  **Evidence gaps and/or recommendations for future research:**  A longitudinal study based on region could look at how the population's attitudes toward vaccines change as parents are exposed to more negative information in the media.  **Source of funding:**  NR |
| 4. | **Researcher(s):**  Azizi et al., 2017  **Title:**  Vaccine hesitancy among parents in a multi-ethnic country, Malaysia  **Year:**  2017  **Journal:**  Vaccine  **Volume:**  35  **Country:**  Malaysia  **WHO Region:**  WPR  **Quality Assessment:**  Include | **What was/were the research question(s):**  1) What is the prevalence of vaccine hesitancy among parents and how does it relate to their socio-demographic characteristics? 2) How can the test-retest reliability of the Parent Attitudes about Childhood Vaccines (PACV) questionnaire be evaluated in Malay?  **What theoretical approach (e.g., Grounded Theory) does the study take (if specified):**  The PACV scale was adopted for this cross-sectional study. | **What population were the sample recruited from:**  Parents attending the Paediatrics and Antenatal clinics of a tertiary hospital in Kuala Lumpur, the capital city of Malaysia.  **How were they recruited:**  Convenience sampling  **How many participants were recruited:**  545  **Were there specific inclusion criteria:**  (a) parents who are at least 18 years old, (b) parents who have at least one child under the age of seven, and (c) a mother who is currently expecting a child.  **Were there specific exclusion criteria:**  Parents from other countries as they may be unfamiliar with Malaysia's vaccination schedule.  **Were there specific vaccines under consideration:**  NR  **Other details:**  When compared to those who already had one or more children, pregnant mothers expecting their first child were nearly four times more likely to be vaccine hesitant. Parents who were unemployed were likewise more likely to be vaccine hesitant. In the multivariate analysis, religion had no significant correlation with vaccine hesitancy. | **Brief description of method and analytical process:**  Cross-sectional study was conducted. Data were analysed using the SPSS software, version 23.0.  **Key outcomes relevant to this review**:  Prevalence rates of parental childhood vaccine hesitancy:  11.6% of parents were found to be vaccine hesitant (PACV score ≥ 50).  Predictors of childhood vaccine uptake or intention (p-value < .05):  Unemployed parents, parents of younger children, non-Muslims, and mothers expecting their first child were substantially more vaccine hesitant than the non-vaccine hesitant group. Other socio-demographic factors such as ethnicity, education level, household income, and gender had no significant influence on vaccine hesitancy.  **How the outcomes were measured:**  The PACV scale was used as the measuring tool. Univariate and multivariate logistic regression analyses were done to evaluate the influence of several vaccine hesitancy determinants such as ethnicity/religion, unemployment and multiparity.  **Potential confounders:**  NR  **Ethical issues:**  The Medical Research Ethics Committee (MREC) of the University of Malaya Medical Centre in Kuala Lumpur, Malaysia, as well as the tertiary hospital's Departments of Paediatrics and Obstetrics & Gynaecology, approved the study. | **Limitations identified by author(s):**  Because the immunisation delays and refusals were self-reported, social desirability bias could be present. The results may be skewed by convenience sampling of participants and data collection in a hospital setting. The authors were unable to assess the validity of the Malay-PACV in their scenario since they did not collect data on actual vaccine uptake. The original PACV, on the other hand, had high validity in other populations and clinical settings.  **Evidence gaps and/or recommendations for future research:**  To avoid the rising trend of vaccine hesitancy, which may eventually lead to vaccine refusal, targeted preventative efforts should be implemented, focusing on the identified high-risk categories.  **Source of funding:**  NR |
| 5. | **Researcher(s):**  Bakhache et al., 2013  **Title:**  Health care providers’ and parents’ attitudes toward administration of new infant vaccines—a multinational survey  NB: As this specific research article has two separate sections (one for healthcare providers and the other for parents), only parental attitudes are considered in the review.  **Year:**  2013  **Journal:**  European Journal of Paediatrics  **Volume:**  172  **Country:**  Multi-national  **WHO Region:**  Multi-regional  **Quality Assessment:**  Include | **What was/were the research questions (relevant to this review):**  1) What are parents' attitudes and perceptions of vaccine efficacy and safety, as well as their general beliefs about childhood vaccinations?  2) What are parents' feelings about co-administering new infant vaccines, such as their preferences for the most vaccine injections per office visit, the variables that influence their decision to vaccinate their child, and their preferences for adding a new vaccine to their child's immunisation schedule?  3) What is the state of meningococcal disease knowledge and attitudes, as well as opinions and perspectives on meningococcal vaccination among parents?  **What theoretical approach (e.g., Grounded Theory) does the study take (if specified):**  The New Vaccinations of Infants in Practice online survey was developed for the study (20 minutes) | **What population were the sample recruited from:**  Parents from seven countries (Australia, Canada, France, Germany, Spain, Sweden, and the UK) who were heavily involved in at least one infant's immunisation decisions between the ages of 0 and 23 months (recruited from Ipsos Healthcare databases)  **How were they recruited:**  Convenience sampling  **How many participants were recruited:**  2460  **Were there specific inclusion criteria:**  (a) Parents ranged in age from 20 to 50 years; (b) there were at least two people living in the respondent's house; (c) there was at least one child aged 0 to 23 months in the respondent's house (parents who had multiple children responded with their youngest child in mind); (d) the parent was completely or heavily involved in making vaccine decisions for their youngest child.; and (e) the child had received or would receive at least one vaccination against any disease.  **Were there specific exclusion criteria:**  Parents who had no child between 0 and 23 months of age.  **Were there specific vaccines under consideration:**  Meningococcal vaccine  **Other details:**  61% of parents were unsure whether their child was at risk for types of meningitis not covered by routine immunisations. Only about 40% of parents knew whether or not their child had had a meningococcal vaccine. | **Brief description of method and analytical process:**  A cross-sectional study was conducted. McNemer’s test and Hierarchical Bayes estimation were used for weighting and statistical analysis.  **Key outcomes relevant to this review**:  Prevalence rates of parental childhood vaccine hesitancy:  16% would not accept the vaccines recommended in the national schedule.  Predictors of childhood vaccine uptake or intention (p-value < .05):  10% of parents thought their child had too many immunisations (needlesticks) during a single appointment. According to 18% of parents, if a vaccine was not on the official schedule, it was not worth getting.  When asked how many vaccine injections parents were okay with their child having during a single medical visit, 15% said one injection and 42% said two injections. When parents were asked what variables influenced their comfort level with their child receiving the maximum number of injections per office visit, 38% expressed concern about "overworking" their child's immune system.  **How the outcomes were measured:**  Online survey instrument, McNemer’s test, Hierarchical Bayes estimation  **Potential confounders:**  NR  **Ethical issues:**  NR | **Limitations identified by author:**  The scenarios in the questionnaire were realistic, but hypothetical, and it was impossible to know whether parents would truly carry out their supposed intentions. As with any survey, the results may be influenced by the respondents' recall bias and response bias. As a result, some survey participants may be tempted to exaggerate their experience or interest in order to please the researcher. Finally, the online survey included parents who had internet access, implying a higher socioeconomic and educational level; this potential selection bias may affect the generalisability of the findings to the entire population.  **Evidence gaps and/or recommendations for future research:**  NR  **Source of funding:**  Novartis Vaccines and Diagnostics, Cambridge, Massachusetts, USA |
| 6. | **Researcher(s):**  Bell et al., 2020  **Title:**  Parents’ and guardians’ views on the acceptability of a future COVID-19 vaccine: A multi-methods study in England  **Year:**  2020  **Journal:**  Vaccine  **Volume:**  38  **Country:**  England  **WHO Region:**  EUR  **Quality Assessment:**  Include | **What was/were the research question(s):**  What are parents’ and guardians’ views on the acceptability of a future COVID-19 vaccine?  **What theoretical approach (e.g., Grounded Theory) does the study take (if specified):**  Mixed-methods approach (online cross-sectional survey and semi-structured interviews developed for the purpose of the study) | **What population were the sample recruited from:**  Parents and guardians (aged 16 and above) who lived in England with a child under the age of 18 months  **How were they recruited:**  Convenience sampling  **How many participants were recruited:**  1252  **Were there specific inclusion criteria:**  1) Parents and guardians who were aged 16 and above, 2) Parents and guardians who said they lived in England, 3) Parents and guardians who had a child under the age of 18 months  **Were there specific exclusion criteria:**  NR  **Were there specific vaccines under consideration:**  COVID-19  **Other details:**  Participants with a lower household income (£35,000) were nearly twice as likely to reject a COVID-19 vaccine for themselves (OR: 2.08, 95% CI: 1.31–3.3) as those with a medium household income (£35,000-£84,999). Participants in the highest income band (>£85,000) were nearly three times as likely to receive the vaccine (OR: 0.35, 95% CI: 0.17–0.73) as those in the intermediate income bracket (£35,000-£84,999). Persons who self-identified as Black, Asian, Chinese, Mixed, or Other ethnicity were 2.7 times (95% CI: 1.27–5.87) more likely than White British, White Irish, or White Other participants to refuse the COVID-19 vaccine. Those who identified their occupation as homemaker were more likely to refuse the immunisation than those who were employed full-time or on parental leave. | **Brief description of method and analytical process:**  Mixed-methods study was conducted. Data analysed using logistic regression analysis, paired samples t-test, Hosmer-Lemeshow test.  **Key outcomes relevant to this review**:  Prevalence rates of parental childhood vaccine hesitancy:  Respondents cited a lack of trust in vaccinations, science, or the medical profession as a reason for refusing to accept a vaccine (4.0% for themselves and 1.6% for their child)  Predictors of childhood vaccine uptake or intention (p-value < .05):  People who self-identified as Black, Asian, Chinese, Mixed, or Other ethnicity were 2.74 times (95% CI: 1.35–5.57) more likely than White British, White Irish, and White Other participants to reject a novel COVID-19 vaccination for their child. Participants in the lowest household income group (£35,000) were 1.8 times (95% CI: 1.17–2.82) as likely to refuse a COVID-19 vaccine for their child as participants in the middle household income bracket (£35,000-£84,999). Participants with more than four children were shown to be four times (OR 4.13; 95% CI: 1.873–9.104) more likely than those with only one child to refuse the immunisation for their children.  **How the outcomes were measured:**  Online questionnaires, Paired samples t-test, Logistic regression analysis, Hosmer-Lemeshow test.  **Potential confounders:**  NR  **Ethical issues:**  Observational Research Ethics Committee of London School of Hygiene & Tropical Medicine approved the study protocol. | **Limitations identified by author:**  The study was conducted during the peak of the COVID-19 epidemic in England, and a poll conducted now that the pandemic has passed its 'peak' of cases and deaths and the lockdown has been lifted may provide different results. The sample was not representative of the general population in terms of household income and race, despite being geographically representative.  **Evidence gaps and/or recommendations for future research:**  Longitudinal studies to assess the acceptability of COVID-19 vaccination over time.  **Source of funding:**  National Institute for Health Research Health Protection Research Unit (NIHR HPRU)) in Immunisation at the London School of Hygiene and Tropical Medicine (LSHTM) in partnership with Public Health England (PHE) |
| 7. | **Researcher(s):**  Bianco et al., 2019  **Title:**  Parent perspectives on childhood vaccination: How to deal with vaccine hesitancy and refusal?  **Year:**  2019  **Journal:**  Vaccine  **Volume:**  37  **Country:**  Italy  **WHO Region:**  EUR  **Quality Assessment:**  Include | **What was/were the research question(s):**  1) What are the attitudes of parents about childhood vaccines and vaccine refusal or delay?  2) What are the roles of the variables mapped as potential determinants?  **What theoretical approach (e.g., Grounded Theory) does the study take (if specified):**  The study, which was cross-sectional in nature, was conducted from April to June 2017. The PACV scale was adopted for the study. | **What population were the sample recruited from:**  Parents with at least one child aged 1–5 years who attended kindergartens in the Catanzaro and Cosenza regions of southern Italy.  **How were they recruited:**  Multi-stage sampling  **How many participants were recruited:**  575  **Were there specific inclusion criteria:**  Parents with at least one kid aged 1–5 years who attended kindergartens in the Catanzaro and Cosenza areas of southern Italy. There were 51 nursery schools in this area, with 900 registered pupils.  **Were there specific exclusion criteria:**  NR  **Were there specific vaccines under consideration:**  NR  **Other details:**  Majority of participants (80.3%) were mothers, with an average age of 37.2 years. Only 63% was employed. The average age of index children was 4.1 years, with males accounting for 50.5%. 68.8% of the parents had two or more children. | **Brief description of method and analytical process:**  Study was cross-sectional. For categorical data, frequencies and percentages were used, whereas for continuous data, mean and standard deviations were used. Variables with p < 0.25 in the univariate analyses were forced into multiple logistic regression models, which were adjusted for potential confounders.  **Key outcomes relevant to this review**:  Prevalence rates of parental childhood vaccine hesitancy:  7.7 % of parents were classified as vaccine hesitant, and 24.6 % reported refusing or delaying at least one vaccine dose for their child.  Predictors of childhood vaccine uptake or intention (p-value < .05):  VH was more likely in parents who chose not to vaccinate their children after receiving information from the media, in those who opposed mandatory vaccinations, and in those who concurred with anti-vax political leaders. Vaccine-refusing/delaying parents were more likely to think that infant vaccinations are primarily a profit-making venture for pharmaceutical firms, and to disagree that only vaccinated children should be permitted to attend kindergarten.  **How the outcomes were measured:**  The PACV scale was used as measuring instrument. To investigate how potential determinants of VH impacted on the dependent variables, the authors employed the t-test (for continuous variables) and Pearson's chi-square (for categorical variables) in the primary analysis.  **Potential confounders:**  Gender, age, marital status, educational level, work activity, and parent's nationality  **Ethical issues:**  The Institutional Ethical Committee (Italy) granted approval to the study protocol. | **Limitations identified by author:**  The cross-sectional design made it impossible to draw conclusions on causality about the observed relationships, and to the self-reporting of practises. The study faced the same difficulty as earlier studies in that parental behaviour was self-reported. Because the data was limited to a region of Southern Italy, there may be concerns regarding the results' generalizability.  **Evidence gaps and/or recommendations for future research:**  Longitudinal studies are required to better characterise trends in the incidence of VH and vaccine refusal or delay.  **Source of funding:**  NR |
| 8. | **Researcher(s):**  Bocquier et al., 2018  **Title:**  Social differentiation of vaccine hesitancy among French parents and the mediating role of trust and commitment to health: A nationwide cross-sectional study  **Year:**  2018  **Journal:**  Vaccine  **Volume:**  38  **Country:**  France  **WHO Region:**  EUR  **Quality Assessment:**  Include | **What was/were the research question(s):**  1) What is the association between parental socioeconomic status (SES) and VH levels?  2) What roles do levels of commitment and trust play in shaping VH?  **What theoretical approach (e.g., Grounded Theory) does the study take (if specified):**  In this study, the 2016 Baromètre santé questionnaire was used, which is a national cross-sectional telephone survey addressing health issues in representative population samples. The French Public Health Agency (Santé publique France) designed and administered the questionnaire. | **What population were the sample recruited from:**  All households with at least one French-speaking person aged 15 to 75.  **How were they recruited:**  Random sampling of households. The study employed an overlapping dual-frame design of landline and mobile phone numbers, which were created at random from prefixes assigned by the electronic communications regulatory authority. For landline phones, one respondent was chosen at random from eligible household members, while for mobile phones, one respondent was chosen at random from eligible regular mobile users.  **How many participants were recruited:**  3927  **Were there specific inclusion criteria:**  All households with at least one French-speaker aged 15 to 75.  **Were there specific exclusion criteria:**  NR  **Were there specific vaccines under consideration:**  Measles, hepatitis B, and HPV  **Other details:**  57% of the respondents were mothers, 49% were under the age of 40, 91% lived with a partner, 68% had one or two children, and 32% had a child under the age of three. | **Brief description of method and analytical process:**  In this cross-sectional study, Chi-square tests were used for bivariate analyses to investigate the relationships between VH levels and respondents' demographic and socioeconomic factors. Then, after controlling for additional sociodemographic factors, the authors used multiple multinomial logistic regression models that included SES and EHI (equivalent household income per month).  **Key outcomes relevant to this review**:  Prevalence rates of parental childhood vaccine hesitancy:  26% of the parents were refusers, 7% delayers, and 13% acceptors with doubts  Predictors of childhood vaccine uptake or intention (p-value < .05):  The prevalence of different VH levels varied significantly depending on parental education. VH was also more prevalent among mothers than fathers, and among parents living with a partner.  **How the outcomes were measured:**  The 2016 Baromètre santé questionnaire was used as the measuring tool. Univariate and multivariate logistic regression analyses were done to evaluate the association between SES and various VH levels.  **Potential confounders:**  NR  **Ethical issues:**  The survey was approved by the French National Commission for Computer Data and Individual Freedom (CNIL). | **Limitations identified by author:**  To begin with, the Baromètre santé survey's cross-sectional nature limits definitive conclusions about the patterns of relationships between VH and its predictors, as well as causality. Second, this study suffers from the standard flaws of quantitative telephone surveys, such as a low response rate (50%). Finally, VH items did not allow for analysis of the reasons why parents chose to refuse or delay a vaccine for their children, as well as the number and type of vaccines they chose.  **Evidence gaps and/or recommendations for future research:**  Future study may find it useful to include additional items linked to other elements of parents' lifestyles. Use of complementary and alternative medicine, organic food consumption, and breastfeeding practises are just a few of such examples.  **Source of funding:**  The Agence Nationale de Sécurité du Médicament et des Produits de Santé (ANSM) and the Agence Nationale de la Recherche (ANR) funded this research. The French government also financed this work through the "Investissements d'avenir" (Investments for the Future) programme, which is handled by ANR. |
| 9. | **Researcher(s):**  Danis et al., 2010  **Title:**  Socioeconomic factors play a more important role in childhood vaccination coverage than parental perceptions: a cross-sectional study in Greece  **Year:**  2010  **Journal:**  Vaccine  **Volume:**  28  **Country:**  Greece  **WHO Region:**  EUR  **Quality Assessment:**  Include | **What was/were the research question(s):**  What are the predictive factors of complete and age-appropriate vaccination status in Greece?  **What theoretical approach (e.g., Grounded Theory) does the study take (if specified):**  Self-administered questionnaire validated in a pilot study | **What population were the sample recruited from:**  Parents of children enrolled in the first year of the Greek Grammar school (about 6 years of age)  **How were they recruited:**  Stratified cluster sampling  **How many participants were recruited:**  3434  **Were there specific inclusion criteria:**  NR  **Were there specific exclusion criteria:**  NR  **Were there specific vaccines under consideration:**  NR  **Other details:**  Of the respondents, 80% were mothers, 19% fathers, with the remainder including grandparents, aunts, uncles and older sisters | **Brief description of method and analytical process:**  Cross-sectional study was conducted. STATA software (Stata Corporation, TX, USA, version 10) was used for data analysis.  **Key outcomes relevant to this review**:  Prevalence rates of parental childhood vaccine hesitancy:  1.5% had negative opinions about vaccines  Predictors of childhood vaccine uptake or intention (p-value < .05):  Being a member of a minority group, having additional siblings, and considering the distance to the immunisation location to be a barrier were all independent predictors of both incomplete and delayed vaccination status. Complete vaccination related to maternal age under 30 years and the belief that natural disease is preferable to vaccine, whereas paternal education of high school or higher was the other independent driver of age-appropriate immunisation. Underimmunisation was explained by socioeconomic factors rather than parental beliefs and attitudes toward vaccination.  **How the outcomes were measured:**  Questionnaire served as measuring instrument. Univariate and multivariate logistic regression models were performed.  **Potential confounders:**  NR  **Ethical issues:**  Ethics Committee of the Institute of Child Health, Athens, Greece approved the study protocol | **Limitations identified by author:**  Due to the low incidence of school attendance, Roma children attending school may not be representative of all Roma children. The same may be said for their parents/guardians, since those who take their children to school are more likely to follow immunisation guidelines. The authors also compared current views and attitudes to those gained from past vaccinations. Because the views were measured after vaccination, the immunisation experiences may have altered some of the attitudes. As a result, if parental opinions were examined prospectively (i.e. before the immunisation began), some of the findings would have been different. They also calculated vaccination coverage among 6-year-olds, who had been immunised nearly 5 years earlier. As a result, the information needed to guide interventions is not as timely as it should be.  **Evidence gaps and/or recommendations for future research:**  NR  **Source of funding:**  NR |
| 10. | **Researcher(s):**  Dasgupta et al., 2018  **Title:**  Vaccine Hesitancy for Childhood Vaccinations in Slum Areas of Siliguri, India  **Year:**  2018  **Journal:**  Indian Journal of Public Health  **Volume:**  62  **Country:**  India  **WHO Region:**  SEAR  **Quality Assessment:**  Include | **What was/were the research question(s):**  What are the proportion and factors contributing to vaccine hesitancy for childhood vaccinations in the slums of Siliguri, India?  **What theoretical approach (e.g., Grounded Theory) does the study take (if specified):**  Study was community-based study with cross-sectional design. A predesigned pretested interview schedule following the pattern of the validated version of the vaccine hesitancy survey tool initially developed by the WHO SAGE working group on vaccine hesitancy was used for the study. | **What population were the sample recruited from:**  Parents of children aged 0–59 months’ residing in slums of Siliguri in 2016  **How were they recruited:**  Cluster sampling  **How many participants were recruited:**  194  **Were there specific inclusion criteria:**  NR  **Were there specific exclusion criteria:**  NR  **Were there specific vaccines under consideration:**  NR  **Other details:**  Most participants were females (58.8%), first birth order (59.3%), nuclear households (51.0%), and lower socioeconomic position (60.3%). 73.7% had completed at least five years of education. | **Brief description of method and analytical process:**  Cross-sectional study was conducted. Binary logistic regression was used to conduct univariate and multivariate analyses.  **Key outcomes relevant to this review**:  Prevalence rates of parental childhood vaccine hesitancy:  83% of parents were vaccine-hesitant  Predictors of childhood vaccine uptake or intention (p-value < .05):  The most common reason for vaccine hesitancy was a desire to avoid vaccination (26.1%). 20.5% were unaware or confused about when and where to get vaccinated; that health care providers did not adequately explain dates and vaccines; and that they had no credible information. The child was unwell or irritable in the other 18.0% of cases. Vaccine hesitancy was shown to be substantially more common among nuclear households, mothers with less than 5 years of schooling, SES Class I, II, and male children, as well as children born in the second or higher birth order.  **How the outcomes were measured:**  Survey instrument, Binary logistic regression (univariate and multivariate analyses)  **Potential confounders:**  NR  **Ethical issues:**  Institutional Ethics Committee approved the study protocol | **Limitations identified by author:**  Aside from the inherent limitations of cross-sectional research, vaccine-specific reasons for hesitancy could not be fully elicited due to the possibility of recall bias.  **Evidence gaps and/or recommendations for future research:**  NR  **Source of funding:**  NR |
| 11. | **Researcher(s):**  Domek et al., 2018  **Title:**  Measuring vaccine hesitancy: Field testing the WHO SAGE Working Group on Vaccine Hesitancy survey tool in Guatemala  **Year:**  2018  **Journal:**  Vaccine  **Volume:**  36  **Country:**  Guatemala  **WHO Region:**  AMR  **Quality Assessment:**  Include | **What was/were the research question(s):**  **What theoretical approach (e.g., Grounded Theory) does the study take (if specified):**  Vaccine Hesitancy Scale (VHS) developed by WHO SAGE Working Group on Vaccine Hesitancy (Spanish version) | **What population were the sample recruited from:**  Parents of infants aged 6 weeks to 6 months who came in for their first wellness visit were registered (March to November 2016).  **How were they recruited:**  Convenience sampling  **How many participants were recruited:**  720  **Were there specific inclusion criteria:**  NR  **Were there specific exclusion criteria:**  NR  **Were there specific vaccines under consideration:**  NR  **Other details:**  Only 0.4% of parents did not believe that vaccines could prevent children from major diseases, and no parent could conceive of a reason why they should not be vaccinated. | **Brief description of method and analytical process:**  Cross-sectional study was conducted. The significance levels in survey differences were assessed using the Chi-square or Fisher's exact test for categorical variables and the ANOVA test for continuous variables. To evaluate the Likert scale questions, factor analysis was used.  **Key outcomes relevant to this review**:  Prevalence rates of parental childhood vaccine hesitancy:  1.1% were vaccine-hesitant  Predictors of childhood vaccine uptake or intention (p-value < .05):  Most parents (59.2%) believed that parents like them do not vaccinate their children with all of the vaccines that are recommended, with more urban parents (69.7% vs. 48.6%; p 0.0001) holding this belief. Time, distance, and cost to get to the clinic, as well as clinic timing and wait time, were found to be important variables preventing immunisation in the urban population when compared to the rural population (12.5% vs. 6.1%; p = 0.0032).  **How the outcomes were measured:**  Chi-square or Fisher's exact test for categorical variables and the ANOVA test for continuous variables. To evaluate the Likert scale questions, factor analysis was used.  **Potential confounders:**  NR  **Ethical issues:**  Study protocol was approved by the Colorado Multiple Institutional Review Board, Universidad del Valle Ethics Committee, and Guatemala National Ethics Committee of the Ministry of Public Health and Social Assistance. | **Limitations identified by author:**  Infants who were presenting for their first wellness check, which included the start of the primary immunisation series, were included in the study. As a result, the sample was skewed toward individuals who were likely to vaccinate, and hesitancy may have been lower than usual. It is also possible that in face-to-face interviews, parents were less likely to admit to vaccine hesitancy than in anonymous questionnaires.  **Evidence gaps and/or recommendations for future research:**  Field testing of the VHS is required in other low- and middle-income countries. It may be useful to track vaccine uptake in the infants studied to see if there is a link between intention and behaviour. To evaluate the validity and reliability of the VHS, more research will be needed in larger groups with higher vaccine hesitancy.  **Source of funding:**  The Eunice Kennedy Shriver National Institute of Child Health & Human Development at the National Institutes of Health |
| 12. | **Researcher(s):**  Dube et al., 2018  **Title:**  Measuring vaccine acceptance among Canadian parents: A survey of the Canadian Immunization Research Network  **Year:**  2018  **Journal:**  Vaccine  **Volume:**  36  **Country:**  Canada  **WHO Region:**  AMR  **Quality Assessment:**  Include | **What was/were the research question(s):**  1) What is the prevalence of vaccine hesitancy among Canadian parents?  2) What factors are associated with the intention of parents to vaccinate their children?  **What theoretical approach (e.g., Grounded Theory) does the study take (if specified):**  The Theory of Planned Behaviour (TPB) was used to examine associations between parents' vaccination knowledge, attitudes, and beliefs and their intention to vaccinate their children in the future. | **What population were the sample recruited from:**  Canadian parents of children aged 24–59 months  **How were they recruited:**  Convenience sampling  **How many participants were recruited:**  2013  **Were there specific inclusion criteria:**  1) Parents from Canada who could read English or French, 2) Parents or caregivers of at least one child between the ages of 24 and 59 months, 3) Parents who had access to the Internet, and 4) Parents who volunteered to take part in a panel study.  **Were there specific exclusion criteria:**  NR  **Were there specific vaccines under consideration:**  NR  **Other details:**  Most of the participants (78.8%) had only one child, ranging in age from 24 to 59 months. 61.5% were women, and a similar proportion (63.1%) were between the ages of 30 and 39. 54% lived in a large city or town. 87.0% were married or in civil partnerships and 68.4% had a college, undergraduate, or graduate degree. | **Brief description of method and analytical process:**  Cross-sectional study, with data collected between 14th to 29^th^ March 2015. To find differences between parents who strongly intended to vaccinate their child in the future and those who had weaker intentions, researchers used univariate analysis with chi-square and independent-sample t tests. A multivariable logistic regression was used to identify predictors of parents' intention to vaccinate their children.  **Key outcomes relevant to this review**:  Prevalence rates of parental childhood vaccine hesitancy:  50.2% had no intention to vaccinate their children in the future  Predictors of childhood vaccine uptake or intention (p-value < .05):  Parents who regularly sought vaccine information, believed it was their job as parents to question vaccines, or who had previously had trouble obtaining vaccination services were less likely to vaccinate their children in the future. Parents who placed a high level of faith in doctors and public health officials were more likely to vaccinate their children. Almost half of the participants (47.8%) said their religious or spiritual beliefs have an impact on their health decisions.  **How the outcomes were measured:**  Univariate analysis with chi-square and independent-sample t tests. Multivariable logistic regression.  **Potential confounders:**  NR  **Ethical issues:**  The study was approved by the research ethics committee at the *Centre de recherche du CHU de Québec – Université Laval*. | **Limitations identified by author:**  An online panel was used, and participants may differ from the general population in terms of specific traits and responses. While the online panel was designed to be representative of the Canadian population in terms of age, region of residence, income, and education, selection bias and non-response bias cannot be eliminated. Other limitations include the lack of actual behavioural outcomes (child vaccination) and the fact that vaccination intention in parents of young children were measured at a time when most recommended infant vaccines should have been administered.  **Evidence gaps and/or recommendations for future research:**  A longitudinal design could provide useful information to better understand what motivates parents to shift from intention to actual vaccination behaviour.  **Source of funding:**  Public Health Agency of Canada and the Canadian Institutes of Health Research |
| 13. | **Researcher(s):**  Dube et al., 2019  **Title:**  Overview of knowledge, attitudes, beliefs, vaccine hesitancy and vaccine acceptance among mothers of infants in Quebec, Canada  **Year:**  2019  **Journal:**  Human Vaccines & Immunotherapeutics  **Volume:**  15  **Country:**  Canada  **WHO Region:**  AMR  **Quality Assessment:**  Include | **What was/were the research question(s):**  What is the level of vaccine hesitancy and vaccination knowledge, attitudes, and beliefs among mothers?  **What theoretical approach (e.g., Grounded Theory) does the study take (if specified):**  Self-administered PACV survey | **What population were the sample recruited from:**  Mothers of newly born infants in four maternity wards in Quebec (Canada)  **How were they recruited:**  Cluster sampling  **How many participants were recruited:**  2645  **Were there specific inclusion criteria:**  In each participating maternity ward, recruitment was limited to mothers aged 18 and above who spoke English or French.  **Were there specific exclusion criteria:**  The study did not include newborns or mothers who required acute care.  **Were there specific vaccines under consideration:**  NR  **Other details:**  At the time of childbirth, most mothers were aged 20–29 years (38.2%) or 30–39 years (56.6%), and the majority were born in Canada (74.3 percent ). M ost of the children were either the mother's first (47.0%) or second (36.3%). More than half of the mothers (55.7%) had a university degree, and the majority (53.6%) were either living with a partner or legally married (37.3%). | **Brief description of method and analytical process:**  In this cross-sectional study, the PACV survey was used to determine VH scores. SAS version 9.4 was used for data analysis.  **Key outcomes relevant to this review**:  Prevalence rates of parental childhood vaccine hesitancy:  15% of mothers had PACV score of 50 or higher. In comparison to 74.1% of mothers who were unsure or did not intend to vaccinate their newborn, nearly half of the mothers (48.9%) who were certain to get their infant vaccinated planned to conduct extensive research before making a final decision (p < 0.0001).  Predictors of childhood vaccine uptake or intention (p-value < .05):  The perceived necessity of vaccinating a child at 2 months of age, a VH score less than 30, anticipated remorse of non-vaccination, and feeling knowledgeable about vaccines were the primary drivers of vaccination intention.  **How the outcomes were measured:**  The PACV survey was used to determine VH scores. To find variables linked to vaccination intention, the researchers used multivariate logistic regression.  **Potential confounders:**  NR  **Ethical issues:**  Each participating facility's Research Ethics Board approved the study protocol. | **Limitations identified by author:**  It is impossible to eliminate selection bias and non-response bias. Younger mothers (under 18), mothers who did not give birth in one of the designated hospitals, and mothers who did not speak English or French were excluded. As with most surveys, there is the possibility of social desirability bias, which means people reply to what they think the researchers want to hear.  **Evidence gaps and/or recommendations for future research:**  NR  **Source of funding:**  The Fonds de recherche du Québec – Santé and the Québec Ministry of Health and Social Services supported this research. |
| 14. | **Researcher(s):**  Fanxing et al., 2020  **Title:**  The determinants of vaccine hesitancy in China: A cross-sectional study following the Changchun Changsheng vaccine incident  **Year:**  2020  **Journal:**  Vaccine  **Volume:**  38  **Country:**  China  **WHO Region:**  WPR  **Quality Assessment:**  Include | **What was/were the research question(s):**  What is the prevalence and determinants of vaccine hesitancy following the Changchun Changsheng vaccine incident (CCVI)?  **What theoretical approach (e.g., Grounded Theory) does the study take (if specified):**  Self-administered PACV survey. The 3C model was used to explore reasons for vaccine hesitancy. | **What population were the sample recruited from:**  Caregivers of all children who visited sampled vaccination  clinics and kindergartens  **How were they recruited:**  Cluster sampling  **How many participants were recruited:**  2124 mothers of children under 6  **Were there specific inclusion criteria:**  NR  **Were there specific exclusion criteria:**  NR  **Were there specific vaccines under consideration:**  NR  **Other details:**  Higher-educated parents who practised Buddhism or other religions were much less likely to vaccinate their children. | **Brief description of method and analytical process:**  Cross-sectional survey. The Wenjuanxing online platform (https://www.wjx.  cn/) and Epidata 3.1 were used for data analyses.  **Key outcomes relevant to this review**:  Prevalence rates of parental childhood vaccine hesitancy:  60% of caregivers were hesitant about getting vaccinated. Among this hesitant population, 26% vaccinated their children with doubts, 31% delayed vaccination, and 3% refused specific vaccines.  Predictors of childhood vaccine uptake or intention (p-value < .05):  Hearing or reading negative information about vaccines (62.2%) and having a terrible experience with past vaccinations for their children (25.3%) were the main reasons for vaccine hesitancy stated by caregivers.  Concerns about vaccine safety among caregivers (24.6%), children's fear of needles (20.6%), indifference about vaccine-preventable diseases (20.5%), and the cost of self-paid vaccines (19.6%) were all significant factors. When compared to 'acceptors with doubts' or 'delayers', 'refusers' reported considerably more loss of confidence, inconvenience, and negative vaccination experiences.  **How the outcomes were measured:**  For categorical data, Chi-square or Fisher's exact tests (if expected frequency < 5) were used, and ANOVA for continuous measures, to evaluate the levels of vaccine hesitancy by participants' demographic characteristics and vaccine confidence. The determinants of vaccine hesitancy were measured using multinomial logistic regression.  **Potential confounders:**  NR  **Ethical issues:**  The study protocol was approved by the Ethics Committees of Fudan University School of Public Health and the London School of Hygiene & Tropical Medicine. | **Limitations identified by author:**  Firstly, the questionnaires were self-administered, which may have resulted in some items being misunderstood. Second, due to the sample approach, there may be some selection bias. Participants were recruited through vaccination clinics, and caregivers who bring their children to clinics for immunisation may be less likely to have vaccine hesitancy than those who do not. As a result, the prevalence of vaccine hesitancy may have been underestimated in the study. Finally, the "refusers" group's small sample size (n = 64) may have diminished the power to identify differences between the refusers and the no hesitancy group. Finally, the researchers did not inquire about caregivers' concerns regarding specific childhood vaccines, and hesitancy levels may differ by vaccine.  **Evidence gaps and/or recommendations for future research:**  A follow-up survey to describe the trend of vaccine hesitancy following the CCVI.  **Source of funding:**  The National Institute for Health Research Health Protection Research Unit (NIHR HPRU) in Immunisation at the London School of Hygiene and Tropical Medicine, in collaboration with Public Health England (PHE), funded this study. |
| 15. | **Researcher(s):**  Frew et al., 2016  **Title:**  Changes in childhood immunization decisions in the United States: Results from 2012 & 2014 National Parental Surveys  **Year:**  2016  **Journal:**  Vaccine  **Volume:**  34  **Country:**  USA  **WHO Region:**  AMR  **Quality Assessment:**  Include | **What was/were the research question(s):**  What are the changes in vaccine decisions made by parents of young children?  **What theoretical approach (e.g., Grounded Theory) does the study take (if specified):**  Web-based national survey based on federal quality requirements (e.g., the CDC-sponsored National Immunization Survey (NIS)), including survey pilot testing with parents of young children via cognitive interviews. | **What population were the sample recruited from:**  Parents of children less than 7 years  **How were they recruited:**  Random sampling  **How many participants were recruited:**  2603 in 2012, 2518 in 2014  **Were there specific inclusion criteria:**  1) Parents and guardians aged ⩾18  2) Parents and guardians living in the U.S.  3) Parents and guardians with children  **Were there specific exclusion criteria:**  NR  **Were there specific vaccines under consideration:**  NR  **Other details:**  In both 2012 and 2014, most respondents were females between the ages of 25 and 44, non-Hispanic whites who were employed, married, had some college degree or more, lived in a metropolitan area, and had a household income of $20,000 to $150,000. | **Brief description of method and analytical process:**  Cross-sectional studies. SPSS version 22.0 and SPSS Complex Sample Module (IBM SPSS Inc., Chicago, IL, USA) were used for statistical analyses.  **Key outcomes relevant to this review**:  Prevalence rates of parental childhood vaccine hesitancy:  In 2012, 5.5% intentionally delayed one or more vaccines, while 5.4% refused one or more vaccines. Refusal rates in 2014 were 5.6% and 3.6%, respectively.  Predictors of childhood vaccine uptake or intention (p-value < .05):  There was no statistically significant difference in intentional and ongoing delay or refusal of any of the recommended childhood vaccinations between 2012 and 2014. All nine non-influenza vaccines had relatively consistent refusal rates. Most parents were more certain of their refusal decisions than of their delay decisions, and they were more certain of their refusal of MMR and chickenpox vaccines than of other vaccines. DTaP, HepB, and PCV vaccines were the least frequently refused.  **How the outcomes were measured:**  Bivariate survey-weighted chi-square analyses were done.  **Potential confounders:**  NR  **Ethical issues:**  The surveys were approved by FHI360 and Westat Institutional Review Boards (IRBs). Studies were also reviewed by the Emory University IRB. | **Limitations identified by author:**  Exclusion of parents less than 18 years, recall bias, response bias, social desirability bias  **Evidence gaps and/or recommendations for future research:**  NR  **Source of funding:**  U.S. Centres for Disease Control and Prevention (CDC) |
| 16. | **Researcher(s):**  Giambi et al., 2018  **Title:**  Parental vaccine hesitancy in Italy – Results from a national survey  **Year:**  2018  **Journal:**  Vaccine  **Volume:**  36  **Country:**  Italy  **WHO Region:**  EUR  **Quality Assessment:**  Include | **What was/were the research question(s):**  What is the prevalence and determinants of vaccine hesitancy among parents of children aged 16–36 months?  **What theoretical approach (e.g., Grounded Theory) does the study take (if specified):**  Self-administered questionnaires as well as online surveys. | **What population were the sample recruited from:**  Parents of children aged 16–36 months, in two categories: 1) an online panel of Italian families, stratified by macro region to mirror the geographic distribution of the reference population (Northern, Central and Southern Italy), and 2) parents visiting paediatricians’ offices and nurseries in five Italian  Regions (Emilia-Romagna, Friuli Venezia Giulia, Marche, Piemonte, and Puglia).  **How were they recruited:**  Stratified sampling  **How many participants were recruited:**  3130  **Were there specific inclusion criteria:**  NR  **Were there specific exclusion criteria:**  NR  **Were there specific vaccines under consideration:**  Tetanus, poliomyelitis, diphtheria, pertussis, *Haemophilus influenzae* type b (Hib), hepatitis B, MMR  **Other details:**  Even though numerous studies have found no increased risk of autism following vaccination, 21% of parents in this study believed that vaccines can cause autism. Furthermore, even though thimerosal is no longer used as a vaccine preservative (except for multidose influenza vaccine), 44% of parents believed that many vaccines contain "mercury." Again, 32% of parents were concerned that vaccination will weaken their child's immune system. | **Brief description of method and analytical process:**  Cross-sectional study. Stata/MP version 13 (Stata Corp, Texas, USA) was used for data analyses.  **Key outcomes relevant to this review**:  Prevalence rates of parental childhood vaccine hesitancy:  15.6% of respondents were vaccine hesitant  Predictors of childhood vaccine uptake or intention (p-value < .05):  1) not receiving a recommendation from a paediatrician to fully vaccinate their child; 2) receiving conflicting vaccination advice; 3) meeting parents of children who had adverse vaccine side effects; and 4) employing non-traditional medical therapies.  **How the outcomes were measured:**  The connection between vaccine hesitancy and socio-demographic variables of parents was investigated using multivariable logistic regression comparing hesitant vs. pro-vaccine parents.  **Potential confounders:**  NR  **Ethical issues:**  NR | **Limitations identified by author:**  The sample's geographic distribution varied slightly from that of the study population. Because of the likely link between hesitancy and area of residence, families residing in north central Italy were over-sampled in several regions, which could have contributed a bias in the estimations.  **Evidence gaps and/or recommendations for future research:**  NR  **Source of funding:**  Italian Ministry of Health |
| 17. | **Researcher(s):**  Gilkey et al., 2016  **Title:**  Vaccination Confidence and Parental Refusal/Delay of Early Childhood Vaccines  **Year:**  2016  **Journal:**  PLoS ONE  **Volume:**  11  **Country:**  USA  **WHO Region:**  AMR  **Quality Assessment:**  Include | **What was/were the research question(s):**  **What theoretical approach (e.g., Grounded Theory) does the study take (if specified):**  The 2011 National Immunisation Survey (NIS) Parental Concerns Module and the Vaccination Confidence Scale were used to assess parents’ vaccination beliefs. | **What population were the sample recruited from:**  Parents of children aged 19 to 35 months. Data was derived from the 2011 NIS, a population-based survey conducted annually by the Centres for Disease Control and Control (CDC).  **How were they recruited:**  Random sampling  **How many participants were recruited:**  9354  **Were there specific inclusion criteria:**  NR  **Were there specific exclusion criteria:**  NR  **Were there specific vaccines under consideration:**  MMR, varicella, seasonal flu  **Other details:**  Male (53%) and female (47%) children were reported by parents in almost similar proportions. Most children were non-Hispanic white (61%) or non-Hispanic black (14%) or Hispanic (15%). Mothers or female guardians made up about three-quarters of the parents (78%). A significant proportion of parents (39%) had a high school diploma or less schooling and lived in poverty (29%). | **Brief description of method and analytical process:**  Cross-sectional survey was conducted. Data were analysed using SAS 9.3 (Cary, NC).  **Key outcomes relevant to this review**:  Prevalence rates of parental childhood vaccine hesitancy:  27% were vaccine-hesitant, 15% were vaccine refusers  Predictors of childhood vaccine uptake or intention (p-value < .05):  Vaccination confidence was linked to early childhood vaccination behaviours, as well as measures of vaccine refusal, vaccine delay and vaccination status. Mean scores on the Vaccination Confidence Scale were strongly associated with these measures, with each one-point increase in mean scale scores corresponding to a reduction in the odds of refusal ranging from 42% for any vaccine to 61% for MMR, and the odds of delay ranging from 19% for any vaccine to 33% for varicella vaccine. These findings show that vaccination confidence is especially important for parents of young children who must make frequent decisions about whether or not to give their children vaccines.  **How the outcomes were measured:**  To determine associations between parents' mean scores on the 8-item Vaccination Confidence Scale and vaccine refusal, vaccine delay, and vaccination status, researchers used multivariable logistic regression models.  **Potential confounders:**  NR  **Ethical issues:**  The University of North Carolina Institutional Review Board approved the study protocol. | **Limitations identified by author:**  The cross-sectional design of this study restricts the capacity to determine the directionality of the connection between vaccination confidence and behaviour. The study's focus on the vaccination beliefs of parents was also a limitation. Although this focus fulfilled the study's primary purpose of validating the Vaccine Confidence Scale, other drivers of early childhood vaccination coverage, such as provider recommendations and clinical systems for patient recall and reminders, are also essential. Another limitation is that the measure of trust was particular to healthcare providers; trust in other entities, such as pharmaceutical corporations or government authorities, as well as trust in vaccinations themselves, may also be relevant. Furthermore, the high mean value for this construct shows a possible ceiling effect, in which respondents gave such high ratings to trust questions that there was little fluctuation.  **Evidence gaps and/or recommendations for future research:**  Future research can develop and evaluate items that better capture variation in parents' faith in their children's immunisation providers, as well as the healthcare system as a whole.  **Source of funding:**  1) The Academic Paediatric Association Young Investigator Award  2. The National Cancer Institute |
| 18. | **Researcher(s):**  Goldman et al., 2020  **Title:**  Caregiver willingness to vaccinate their children against COVID-19: Cross sectional survey  **Year:**  2020  **Journal:**  Vaccine  **Volume:**  38  **Country:**  Multi-national  **WHO Region:**  Multi-regional  **Quality Assessment:**  Include | **What was/were the research question(s):**  What are the predictors associated with caregivers’ intention to vaccinate their children against COVID-19?  **What theoretical approach (e.g., Grounded Theory) does the study take (if specified):**  Studies were conducted as part of the COVID-19 Parental Attitude Study  (COVIPAS) of parents and caregivers seeking emergency care for their children during the COVID-19 era. Survey tool was available in English, French, German, Spanish, Japanese, Italian, and Hebrew. | **What population were the sample recruited from:**  Caregivers bringing their children to 16 paediatric Emergency Departments (ED) across six nations from March 26 to May 31, 2020  **How were they recruited:**  Convenience sampling  **How many participants were recruited:**  1541  **Were there specific inclusion criteria:**  NR  **Were there specific exclusion criteria:**  NR  **Were there specific vaccines under consideration:**  COVID-19  **Other details:**  The median age of the children was 7.5 (SD = 5.0) years, and the caregivers' median age was 39.9 (SD = 7.6) years. Parents (97.5%) completed most of the surveys, as opposed to other caregivers. 12% had children with a chronic condition, and 15% had a probable contraindication to live vaccinations (such as cancer or being on immunosuppressive medicines). 21 % of the surveys were completed in the United States, 35% in Canada, 28% in Switzerland, 8% in Spain, 6% in Israel, and 2% in Japan. | **Brief description of method and analytical process:**  Cross-sectional study across six countries. Univariate analysis was used to determine which factors were significantly linked to the decision to vaccinate children: the Mann-Whitney test for non-normal continuous variables, the independent t-test for normally distributed continuous variables, and the Chi-square or Fisher's exact test for categorical variables. Using all of the variables that indicated significance (p < 0.1) in the univariate analysis, the researchers conducted multivariable logistic regression analysis to obtain the adjusted odds ratio of agreeing to vaccinate children.  **Key outcomes relevant to this review**:  Prevalence rates of parental childhood vaccine hesitancy:  33% had no intention to vaccinate their children against COVID-19  Predictors of childhood vaccine uptake or intention (p-value < .05):  Several factors were associated with vaccination intention, including children who were older, children without a chronic illness, when fathers completed the survey, children up-to-date on their vaccination schedule, recent history of influenza vaccination, and caregivers concerned their child had COVID-19 at the time of survey completion in the ED all had higher intended uptake. The most common reason given by caregivers for wanting to vaccinate their children was to protect them (62%), while the most common reason given by caregivers for refusing immunisation was the vaccine's novelty (52%).  **How the outcomes were measured:**  Univariate analysis (Mann-Whitney test, independent t-test, Chi-square test, Fisher's exact test) and multivariable logistic regression analysis  **Potential confounders:**  NR  **Ethical issues:**  The Institutional Review Boards (IRB) of participating countries approved the study protocol. | **Limitations identified by author:**  First, because the survey was conducted in a hospital ED during the peak of COVID-19, the population of parents and other caregivers who responded is not representative of all caregivers in the six countries where it was conducted. In addition, requiring a smartphone to take the poll may prevent some people from taking part.  Second, caregivers discussed their concerns about vaccinating their child at a period of high uncertainty (no school, work-at-home), and their willingness to vaccinate against COVID-19 may differ when community life returns to normal and the number of infected patients decreases. Finally, the researchers developed a hypothetical vaccine, which, once available and tested, may provide caregivers with fresh knowledge that causes them to reconsider their decision to vaccinate their children.  **Evidence gaps and/or recommendations for future research:**  Future study should take regional and geographic disparities into account, particularly when determining parental reasons for not planning to vaccinate their children.  **Source of funding:**  NR |
| 19. | **Researcher(s):**  Greenberg et al., 2017  **Title:**  Vaccine Hesitancy: In Search of the Risk Communication Comfort Zone  **Year:**  2017  **Journal:**  PLoS Currents  **Volume:**  9  **Country:**  Canada  **WHO Region:**  AMR  **Quality Assessment:**  Include | **What was/were the research question(s):**  What are parental attitudes regarding childhood vaccinations in Canada?  **What theoretical approach (e.g., Grounded Theory) does the study take (if specified):**  The Angus Reid Forum Panel, a prominent consumer panel of over 150,000 Canadian adults aged 18 and older spread throughout all geographic regions of Canada, was used to administer closed and open-ended questions in November 2015. | **What population were the sample recruited from:**  Canadian parents of children aged 5 and younger  **How were they recruited:**  The Angus Reid Online Forum panel was used to recruit eligible participants.  **How many participants were recruited:**  1,000  **Were there specific inclusion criteria:**  Parents with vaccine-aged children  **Were there specific exclusion criteria:**  NR  **Were there specific vaccines under consideration:**  MMR  **Other details:**  Most parents (57%) prefer to get their health news and information from online sources (e.g., Google, social media, websites, etc.), followed by television or radio (29%). A very small percentage (5%) said they frequently use scientific sources, such as medical journals, to navigate the ever-changing health information landscape. | **Brief description of method and analytical process:**  Cross-sectional survey was conducted. Online survey consisted of 25 questions split into 4 main sections: perceptions regarding vaccines and vaccination; perspectives on the public debate about vaccinations and vaccine-preventable disease; information seeking needs and practises, including media usage and trust in institutional sources; and communication techniques.  **Key outcomes relevant to this review**:  Prevalence rates of parental childhood vaccine hesitancy:  A significant proportion of parents believe or are unsure whether there is a link between vaccines and autism (28%), are concerned that vaccines may cause major harm to their children (27%), or believe the pharmaceutical industry is driving the campaign for mandatory immunisation (33%).  Predictors of childhood vaccine uptake or intention (p-value < .05):  Even though more than 90% of parents have had their children vaccinated, 44% believe vaccination should remain a personal choice (49% disagreed, and 7% were unsure). When asked if, except for medical exemptions, schools and day-care institutions should deny children who have not been vaccinate, about 65% of respondents replied affirmatively, and 66% agreed that "parents who do not have their children immunised (except in cases involving medical exemptions) are irresponsible." Furthermore, while most parents have had their children vaccinated and many feel that there is scientific consensus on vaccine safety and effectiveness, only 33% agree that "drug companies are behind the government's push for mandatory vaccination."  **How the outcomes were measured:**  NR  **Potential confounders:**  NR  **Ethical issues:**  NR | **Limitations identified by author:**  First, while the online panel for the survey was designed to be representative of the Canadian population in terms of age, residence region, income, and education, selection bias and non-response bias could not be entirely out. Second, parents self-reported their child's MMR vaccination decision, which could lead to recall bias, and there was no additional measure in the study to examine parental vaccine hesitancy attitudes throughout the spectrum. As a result, because most parents said their child had been vaccinated, their reflections on the standard public health messages used to persuade parents about the benefits of vaccination, as well as any suggestions made by parents that might be persuasive in encouraging parents to vaccinate their children, cannot be expected to be effective specifically for vaccine hesitant parents.  **Evidence gaps and/or recommendations for future research:**  Future research should build on these findings by putting risk communication strategies to the test with parents who fall on different sides of the vaccine hesitancy spectrum.  **Source of funding:**  Canadian Immunization Research Network and Canadian Institutes for Health Research and Public Health Agency of Canada. |
| 20. | **Researcher(s):**  Hak et al., 2005  **Title:**  Negative attitude of highly educated parents and health care workers towards future vaccinations in the Dutch childhood vaccination program  **Year:**  2005  **Journal:**  Vaccine  **Volume:**  23  **Country:**  The Netherlands  **WHO Region:**  EUR  **Quality Assessment:**  Include | **What was/were the research question(s):**  What are the attitudes of parents towards possible future vaccinations for their children, as well as the behavioural variables linked to a negative attitude?  **What theoretical approach (e.g., Grounded Theory) does the study take (if specified):**  Focus group session explored possible determinants of parental attitudes. Outcome of the discussion informed the development of a questionnaire, of which most questions were based on determinants of health behaviour outlined by the Health Belief Model (HBM). | **What population were the sample recruited from:**  Parents of children aged between 3 months and 5 years attending day-care centres  **How were they recruited:**  NR  **How many participants were recruited:**  283  **Were there specific inclusion criteria:**  NR  **Were there specific exclusion criteria:**  NR  **Were there specific vaccines under consideration:**  NR  **Other details:**  According to 89% of respondents, there is a critical need to improve current parental health education. Most respondents (47%) prefer oral education from health-care providers, whereas 27% prefer receiving an information booklet at home and 14% prefer educational television programmes. | **Brief description of method and analytical process:**  Mixed-methods study involving polytomous logistic regression analysis.  **Key outcomes relevant to this review**:  Prevalence rates of parental childhood vaccine hesitancy:  One-tenth (11%) had no intention of complying with any new immunisation.  Predictors of childhood vaccine uptake or intention (p-value < .05):  A high education level of the parent, being a health care worker, lack of religion, perception of vaccine ineffectiveness, and the belief that vaccinations cause asthma or allergies.  **How the outcomes were measured:**  Univariate and multivariate logistic regression analysis  **Potential confounders:**  NR  **Ethical issues:**  NR | **Limitations identified by author:**  The study was subjected to response bias. When compared to the Dutch parental population, the percentage of highly educated parents who participated in the study (48% against 30%) and health care employees (18% versus 8%) was higher, which increased the statistical power to detect statistically significant relationships. Finally, the research was probably insufficient in detecting statistically significant relationships with rare determinants. For example, none of the participants were orthodox-reformed (a religious group that is known to oppose vaccination in general) and there were no parents from non-European nations. As a result, it is unclear if the findings could be applied to these groups.  **Evidence gaps and/or recommendations for future research:**  NR  **Source of funding:**  University Medical Centre Utrecht |
| 21. | **Researcher(s):**  Henrikson et al, 2017  **Title:**  Longitudinal Trends in Vaccine Hesitancy in a Cohort of Mothers Surveyed in Washington State, 2013-2015  **Year:**  2017  **Journal:**  Public Health Reports  **Volume:**  132  **Country:**  USA  **WHO Region:**  AMR  **Quality Assessment:**  Include | **What was/were the research question(s):**  How does parental vaccine hesitancy change among parents as their children age?  **What theoretical approach (e.g., Grounded Theory) does the study take (if specified):**  Mothers were surveyed at their baby’s birth, age 6 months, and age 24 months using the validated PACV tool | **What population were the sample recruited from:**  Mothers of 24-month-old children who participated in a two-arm clinic-level cluster randomised study that was completed in Washington State in 2013.  **How were they recruited:**  Participants in original trial were readministered the PACV when their child was 24 months old.  **How many participants were recruited:**  237  **Were there specific inclusion criteria:**  NR  **Were there specific exclusion criteria:**  NR  **Were there specific vaccines under consideration:**  NR  **Other details:**  The mean age was 32.3 years, 78.5% of the population were college educated, and 80.5% were white. Respondents were more likely to be married, to have a greater income, and to have a better level of education compared to all mothers who took part in the study. | **Brief description of method and analytical process:**  Longitudinal study  **Key outcomes relevant to this review**:  Prevalence rates of parental childhood vaccine hesitancy:  9.7% were vaccine-hesitant compared to 13.6% at baseline  Predictors of childhood vaccine uptake or intention (p-value < .05):  Fear that a vaccine might have a serious adverse effect in a child ((42.2% at baseline to 33.8% at 24 months, P = .12), concern that childhood shots might not be safe (34.2% to 24.9%, P = .26), concern that a vaccine would not prevent the disease (27.4% to 22.8%, P = .13), belief that children receive more shots than they need ((16.5% to 13.1%, P = .07).  **How the outcomes were measured:**  PACV survey instrument, Friedman test, linear regression analysis  **Potential confounders:**  NR  **Ethical issues:**  NR | **Limitations identified by author:**  Limitations included the use of a complete-case strategy, which excluded mothers who did not complete all surveys. Sensitivity studies that included data from all mothers who completed a baseline survey, on the other hand, yielded comparable results.  **Evidence gaps and/or recommendations for future research:**  Replication of the research findings in maternal populations from various geographic regions.  **Source of funding:**  The Group Health Foundation |
| 22. | **Researcher(s):**  Hu et al., 2019  **Title:**  Measuring childhood vaccination acceptance of mother in Zhejiang province, East China  **Year:**  2019  **Journal:**  Human Vaccines & Immunotherapeutics  **Volume:**  15  **Country:**  China  **WHO Region:**  WPR  **Quality Assessment:**  Include | **What was/were the research question(s):**  What is the prevalence of vaccine hesitancy and the risk factors associated with mother’s intention to vaccinate in Zhejiang province, China?  **What theoretical approach (e.g., Grounded Theory) does the study take (if specified):**  Attitudes toward vaccination, perceived social support, perceived behavioural control, and mothers' intention to immunise their children were all constructed using the Theory of Planned Behaviour (TPB). Vaccine hesitancy was estimated using questionnaire developed by Zhejiang provincial centre for disease control and prevention (ZJCDC)) (~15 minutes). | **What population were the sample recruited from:**  Mothers of children aged 24–35 months in Zhejiang province, China  **How were they recruited:**  **How many participants were recruited:**  770  **Were there specific inclusion criteria:**  NR  **Were there specific exclusion criteria:**  NR  **Were there specific vaccines under consideration:**  NR  **Other details:**  Most mothers were under 30 years old (63.5%), had a senior middle school or higher degree of education (82.5%), and worked (70.6%). 53.1% lived in rural areas (53.1%). 61.9% were residents, and a similar proportion had a monthly income of 5000–10,000 CNY (64.4%). 8.1% had more than three children. | **Brief description of method and analytical process:**  Cross-sectional study was conducted. Univariate and multivariate analyses were used to compare mothers with strong intentions to immunise their children in the future to mothers with weaker intentions.  **Key outcomes relevant to this review**:  Prevalence rates of parental childhood vaccine hesitancy:  12.7% had weak intentions to vaccinate their children.  Predictors of childhood vaccine uptake or intention (p-value < .05):   A small proportion had difficulty getting a vaccination appointment due to inconvenient work hours (15.2%) or finding time to phone the clinic (11.2%).19.5% reported that vaccination was inconvenient and/or that waiting time at the clinic was unreasonable, and 24.9% did not know when immunisations were needed.  **How the outcomes were measured:**  Mothers with strong intentions to immunise their children in the future were compared to mothers with weaker intentions using univariate and multivariate analyses.  **Potential confounders:**  NR  **Ethical issues:**  The ethical review board of ZJCDC approved the manuscript. | **Limitations identified by author:**  First, because only two items were included, the internal consistency of the two TPB constructs was minimal. Second, the intention to vaccinate children was assessed at the time when most of the recommended vaccinations were scheduled to be given. Third, face-to-face interview was used to develop the study. As a result, potential recall and social desirability bias may not be ruled out, affecting the findings.  **Evidence gaps and/or recommendations for future research:**  NR  **Source of funding:**  NR |
| 23. | **Researcher(s):**  Kalok et al., 2020  **Title:**  Vaccine hesitancy towards childhood immunisation amongst urban pregnant mothers in Malaysia  **Year:**  2020  **Journal:**  Vaccine  **Volume:**  38  **Country:**  Malaysia  **WHO Region:**  WPR  **Quality Assessment:**  Include | **What was/were the research question(s):**  What is the prevalence of vaccine hesitancy amongst urban pregnant mothers?  **What theoretical approach (e.g., Grounded Theory) does the study take (if specified):**  Self-administered PACV questionnaire | **What population were the sample recruited from:**  Women who received their prenatal care at a teaching hospital in Kuala Lumpur  **How were they recruited:**  Convenience sampling  **How many participants were recruited:**  1081  **Were there specific inclusion criteria:**  Women who were pregnant and had at least one child older than one year  **Were there specific exclusion criteria:**  1) Women who were pregnant and had a miscarriage or a foetal congenital abnormality.  2) Mothers who failed or postponed vaccinations for their infants due to a lack of vaccines, limited access to vaccines, or medical reasons such as immunosuppression.  **Were there specific vaccines under consideration:**  NR  **Other details:**  Most of the expectant mothers in this study (64.1%) were over 30 years old. Most (78.7%) were Malay and Muslim (80.3%). More than two-thirds (76.5%) had a diploma or higher educational degree, and over 80% were employed. A little more than half of them had only one child. | **Brief description of method and analytical process:**  Cross-sectional study. SPSS (IBM SPSS Statistics, Version 24.0 Armonk, NY: IBM Corp) was used for data analysis  **Key outcomes relevant to this review**:  Prevalence rates of parental childhood vaccine hesitancy:  8% of participants were vaccine hesitant  Predictors of childhood vaccine uptake or intention (p-value < .05):  Vaccine hesitancy was linked to ethnicity, religion, the number of children, educational level, and employment status. Non-Malay and non-Muslim mothers were more likely to be vaccine hesitant. Vaccine hesitancy was less common among employed pregnant women and those with monthly salaries surpassing MYR2000. Those with more than one child were similarly less likely to be hesitant.  **How the outcomes were measured:**  The PACV Survey was used to assess vaccine hesitancy in both English and validated Malay versions. Using bivariate and multivariate logistic regression, the association between demographic variables, information source and vaccine hesitancy was analysed.  **Potential confounders:**  NR  **Ethical issues:**  University of Kuala Lumpur (UKM) Medical Research and Ethics Committee | **Limitations identified by author:**  Selection bias may have been caused by convenience sampling and data collection in a hospital setting. Because the teaching hospital was largely sponsored by the government, the price of consultation and treatment may be prohibitive for those with lesser incomes. The fact that over 75% of the women had a tertiary education suggests that the study cohort was made up of highly educated women. As a result, the results may not accurately reflect the genuine level of vaccine hesitancy among Malaysia's urban population.  **Evidence gaps and/or recommendations for future research:**  A multicentre study will provide a more accurate depiction of the research population. The goal of the cross-sectional study was to determine whether there was an association rather than a causal relationship. A longitudinal study could be useful in determining if vaccination hesitancy leads to childhood vaccine refusal, particularly in new-borns. Because the PACV is designed to assess immunisation behaviour through past vaccination of existing children, the researchers solely used multiparous women in their study. As a result, they lacked information on primigravidas. Future research into vaccine hesitancy among nulliparous women will necessitate PACV score adjustments.  **Source of funding:**  UKM |
| 24. | **Researcher(s):**  Khattak et al., 2020  **Title:**  Prevalence of Parental refusal rate and its associated factors in routine immunization by using WHO Vaccine Hesitancy tool: A Cross sectional study at district Bannu, KP, Pakistan  **Year:**  2020  **Journal:**  International Journal of Infectious Diseases  **Volume:**  104  **Country:**  Pakistan  **WHO Region:**  EMR  **Quality Assessment:**  Include | **What was/were the research question(s):**  1) What is the vaccination refusal rate of parents who refuse routine immunisation for their children?  2) What are the associated factors and perceptions of parents who refuse routine immunisation for their children?  **What theoretical approach (e.g., Grounded Theory) does the study take (if specified):**  Self-administered survey instrument (WHO SAGE WG Vaccine Hesitancy Tool) | **What population were the sample recruited from:**  Parents with children aged 0–59 month who have at least 12 months of residence in Bannu District, Khyber Pakhtunkhwa Province, Pakistan.  **How were they recruited:**  Multi-stage cluster sampling  **How many participants were recruited:**  610  **Were there specific inclusion criteria:**  1) Parents with children aged 0–59 months; and 2) Parents with at least 12 months of residence in the chosen geographic area.  **Were there specific exclusion criteria:**  Parents who were involved in the delivery of any form of health service, notably healthcare workers  **Were there specific vaccines under consideration:**  Polio  **Other details:**  Mothers were less likely than fathers to own a mobile phone (14.1% vs 89.4%), and most of the hesitant population had minimal education (85.3%). The immunisation rejection rate was greater in parents with food security (51.8%) than in parents with mild food insecurity (36.5%) or high food insecurity (11.8%). Fathers with a high degree of education and a job were less likely to reject vaccinations for their children. | **Brief description of method and analytical process:**  Cross-sectional survey. SPSS Version 22 (IBM Corp., Armonk, NY, USA) was used for data analysis.  **Key outcomes relevant to this review**:  Prevalence rates of parental childhood vaccine hesitancy:  27.9% of parents were vaccine refusers  Predictors of childhood vaccine uptake or intention (p-value < .05):  Parental vaccine rejection was found to be linked to parental education, employment status, household income and food insecurity. There was no link between parental immunisation refusal and gender, age, possession of certain amenities, or depression. Most parents who declined to have their children vaccinated believed that vaccination had substantial side effects. As a result, 19.4% of parents disagreed with doctors' advice that their children be vaccinated. The statement that vaccination can safeguard children was disputed by more than half of parents (50.6%).  **How the outcomes were measured:**  The World Health Organization (WHO) SAGE Working Group on Vaccine Hesitancy Survey Tool. For associations, logistic regression was used, and multi-regression was used to identify potential confounders.  **Potential confounders:**  NR  **Ethical issues:**  The Institutional Review Board and Ethics Committee of Khyber Medical University, Peshawar approved the study protocol. | **Limitations identified by author:**  Because it was impossible to interview mothers due to cultural constraints, only fathers were surveyed. Also, it was difficult to evaluate if polio campaigns have a positive or negative effect on vaccination rejection (polio campaigns for children aged 5 years are held virtually every month in Pakistan); it is still unclear whether polio campaigns have a positive or negative effect on vaccination refusal. Finally, due to the cross-sectional study design, it was impossible to demonstrate causal relationships between parental vaccination refusal status and any of the independent factors.  **Evidence gaps and/or recommendations for future research:**  NR  **Source of funding:**  NR |
| 25. | **Researcher(s):**  Larson et al., 2015b  **Title:** Measuring Vaccine Confidence: Introducing a Global Vaccine Confidence Index **Year:**  2015  **Journal:**  PLoS Currents  **Volume:**  7  **Country:**  Multi-national  **WHO Region:**  Multi-regional  **Quality Assessment:**  Include | **What was/were the research question(s):**  What is the level of parental trust in vaccines and immunisation programmes from a global perspective?  **What theoretical approach (e.g., Grounded Theory) does the study take (if specified):**  The Vaccine Confidence Index (VCI) was developed by the researchers, taking cues from other social science tools such as the Consumer Confidence Index (CCI) that measure confidence more generally, placing a finger on the pulse of a set of public sentiments, which influence vaccination behaviours, with consequences for the whole population. The sentiments measured by the VCI are trust in vaccination and the entities with whom it is linked, and vaccine sentiments, like the sentiments evaluated by the CCI, are influenced by broader social dynamics. | **What population were the sample recruited from:**  Georgian, Indian, Nigerian, Pakistani, and British parents of children under the age of five  **How were they recruited:**  Stratified/Random sampling  **How many participants were recruited:**  Georgia (n=1000); India (n=1259); Pakistan (n=2609); UK (n=2055); Nigerian households (n=12554); Nigerian health providers (n=1272)  **Were there specific inclusion criteria:**  NR  **Were there specific exclusion criteria:**  NR  **Were there specific vaccines under consideration:**  NR  **Other details:**  In comparison to the other nations studied, the UK sample had less respondents with children under the age of five (RCU5s). When compared to RCU5s from other nations, UK RCU5s were more hesitant to vaccinate. In Georgia, however, hesitant parents made up a smaller percentage of RCU5s, but most of those who hesitated did not receive the vaccine. In all nations except India, RCU5s were more likely to believe that all or most people in their community get their children vaccinated, and less likely to indicate they "don't know" how many get their children vaccinated, compared to respondents who did not have children under the age of five. | **Brief description of method and analytical process:**  Country-specific data collection methods were employed, including random sampling, stratified sampling, face-to-face in-house interviews, online interviews, computer-assisted telephone interviewing (CATI), computer assisted personal interviewing (CAPI)  **Key outcomes relevant to this review**:  Prevalence rates of parental childhood vaccine hesitancy:  Georgia had the largest percentage of vaccine refusers (60%) among those who reported hesitancy, followed by Nigeria, where 22.7% of hesitancy-reporting families refused immunisation.  Predictors of childhood vaccine uptake or intention (p-value < .05):  **How the outcomes were measured:**  The researchers developed the Vaccine Confidence Index as a measuring tool.  **Potential confounders:**  NR  **Ethical issues:**  NR | **Limitations identified by author:**  First, survey techniques differed slightly between countries. In Nigeria, for example, data was gathered through face-to-face interviews done within houses, whereas in the United Kingdom, the survey was completed online. Responses could have been influenced by the different forms. Even though the same fundamental questions were asked in each of the five countries, the whole collection of survey questions was not identical in each. This limited the range of comparisons feasible, as surveys in India and the United Kingdom, for example, queried respondents about their confidence in emergency services, but not in other nations. Furthermore, the local team in Georgia did not include questions about trust in immunisation programmes or other health services, so this phenomenon could not be compared. In Georgia, respondents were also questioned if they had children under the age of 15, as compared to the age of five in the other nations examined. These factors could lead to unaccounted-for differences in findings among countries.  **Evidence gaps and/or recommendations for future research:**  NR  **Source of funding:**  NR |
| 26. | **Researcher(s):**  Musa et al., 2019  **Title:**  Vaccine hesitancy among parents in Kuala Lumpur: a single centre study  **Year:**  2019  **Journal:**  F1000 Research  **Volume:**  8  **Country:**  Malaysia  **WHO Region:**  WPR  **Quality Assessment:**  Include | **What was/were the research question(s):**  1) What is the prevalence of VH among parents in Kuala Lumpur, Malaysia?  2) What are the predictors associated with a VH attitude in parents in Kuala Lumpur, Malaysia?  **What theoretical approach (e.g., Grounded Theory) does the study take (if specified):**  Self-administered PACV survey (10-15 minutes) | **What population were the sample recruited from:**  Parents attending Tanglin Health Community Clinic, Kuala Lumpur  **How were they recruited:**  Convenience sampling  **How many participants were recruited:**  337  **Were there specific inclusion criteria:**  1) competent to speak and interpret English or Malay languages; 2) aged 20 or older; and 3) provided written consent.  **Were there specific exclusion criteria:**  1) being too unwell or unsettled to complete the questionnaire, and 2) not being Malaysian citizens.  **Were there specific vaccines under consideration:**  NR  **Other details:**  Females made up 60.2% of the participants. In comparison to all other ethnic groups, Malays accounted for 80.3%. | **Brief description of method and analytical process:**  Cross-sectional study.  **Key outcomes relevant to this review**:  Prevalence rates of parental childhood vaccine hesitancy:  14.5% of respondents  Predictors of childhood vaccine uptake or intention (p-value < .05):  1) The introduction of a new vaccine, 2) Previous negative vaccination experiences, 3) Mistrust of pharmaceutical firms, 4) Mistrust of health institutions and healthcare providers, and 5) Being male  **How the outcomes were measured:**  PACV scale; univariate analysis; multivariate analysis  **Potential confounders:**  NR  **Ethical issues:**  The Human Research Ethics Committee of Monash University and the Medical Research Ethics Committee of the Malaysian Ministry of Health approved the study protocol. | **Limitations identified by author:**  Firstly, the study design eliminated the possibility of causality between the variables examined for VH. Second, the study's findings are dependent on the parent's self-perception at a specific point in time. This could change depending on the time, place, and situation.  Third, there were some missing data that could not be accounted for in the study since the participants were given the option of whether or not to answer each question. It is unclear whether this was due to a lack of understanding of the questions or other factors.  Fourth, participants were only included if they could read and understand Malay or English. This resulted in selection bias, impacting on the results because it left out other Malaysian languages like Mandarin and Tamil. Fifth, parents were recruited using convenience sampling, which resulted in a highly skewed ethnic and religious group, with Malays and Muslims accounting for over 90% of the population. Because this does not represent the entire Malaysian population, the findings cannot be applied to all Malaysian parents. To obtain a more generalisable sample, a simple random sampling method could be used to recruit participants.  Finally, this research is based in a specific location in Kuala Lumpur's urban area. In a semi-urban or rural population, the responses and outcomes may differ.  **Evidence gaps and/or recommendations for future research:**  Other elements that may be associated with VHPs in a multi-ethnic country, such as a link between cultural or social differences and VH in parents, should be investigated further. To investigate this knowledge gap in a varied population, a qualitative research design may be required.  **Source of funding:**  Global Public Health Polling Network |
| 27. | **Researcher(s):**  Napolitano et al., 2018  **Title:**  Investigating Italian parents’ vaccine hesitancy: A cross-sectional survey  **Year:**  2018  **Journal:**  Human Vaccines & Immunotherapeutics  **Volume:**  14  **Country:**  Italy  **WHO Region:**  EUR  **Quality Assessment:**  Include | **What was/were the research question(s):**  What is the prevalence of vaccine hesitancy among parents and its associated factors?  **What theoretical approach (e.g., Grounded Theory) does the study take (if specified):**  Self-administered PACV survey (Italian version) | **What population were the sample recruited from:**  Parents of children aged 2 to 6 years old who attend five pre-schools in the Naples area  **How were they recruited:**  Cluster sampling  **How many participants were recruited:**  437  **Were there specific inclusion criteria:**  NR  **Were there specific exclusion criteria:**  NR  **Were there specific vaccines under consideration:**  NR  **Other details:**  The participants were mostly female (82.1%) and married (86.2%), with an average age of 37.1 years, almost half had a high school education (46.9%), more than half were employed (60.5%), and 69.2% had more than one child. More than half of the parents (53.8%) desired more information regarding their children's vaccines. | **Brief description of method and analytical process:**  Cross-sectional study. Stata version 10.1 was used for data analysis.  **Key outcomes relevant to this review**:  Prevalence rates of parental childhood vaccine hesitancy:  34.7% of parents were vaccine hesitant (PACV score ≥50)  Predictors of childhood vaccine uptake or intention (p-value < .05):  Vaccine hesitancy was more common among parents concerned about vaccine side effects and safety. None of the respondents' sociodemographic characteristics were found to be significant determinants. Parents who did not trust paediatricians were more hesitant.  Furthermore, more than half of the participants required more knowledge about childhood immunisations, with parents of first-born children being more affected by this outcome compared to parents of second-born children.  **How the outcomes were measured:**  PACV scale; univariate analysis; multivariate analysis  **Potential confounders:**  NR  **Ethical issues:**  Ethics Committee of the Teaching Hospital of the University of Campania “Luigi Vanvitelli” approved the study protocol. | **Limitations identified by author:**  Firstly, the researchers were unable to draw any conclusions about the causal relationship between VH variables due to the cross-sectional study design. Second, there is a risk of recall bias in all research based on retrospective data obtained using self-administered questionnaires and not confirmed by medical records, because participants may not remember or report certain events. Third, because some of the study's volunteers may have been sensitive to certain topics, the replies may not be objective and may differ from their genuine feelings. As a result, there is a risk of overestimation of vaccination coverage as well as the possibility of social desirability bias. Finally, the fact that mothers made up most of the sample could be considered a shortcoming.  **Evidence gaps and/or recommendations for future research:**  NR  **Source of funding:**  NR |
| 28. | **Researcher(s):**  Opel et al., 2011b  **Title:**  Validity and reliability of a survey to identify vaccine-hesitant parents  **Year:**  2011  **Journal:**  Vaccine  **Volume:**  29  **Country:**  USA  **WHO Region:**  AMR  **Quality Assessment:**  Include | **What was/were the research question(s):**  What is the construct validity and reliability of the Parent Attitudes about Childhood Vaccines (PACV) survey?  **What theoretical approach (e.g., Grounded Theory) does the study take (if specified):**  Self-administered PACV questionnaire (5-7 minutes) | **What population were the sample recruited from:**  Parents of 19–35-month-old children in a closed model Health Maintenance Organisation (HMO)  **How were they recruited:**  Simple random sampling  **How many participants were recruited:**  230  **Were there specific inclusion criteria:**  1) English-speaking parents 2) Parents ≥18 years old 3) Parents had children aged 19-35 months old singleton children born between April 2007 and August 2008 4) Parents belonged to a large, integrated, US health care delivery system (Group Health Cooperative (GHC), Seattle, Washington  **Were there specific vaccines under consideration:**  NR  **Were there specific exclusion criteria:**  NR  **Other details:**  Children with a parent who was 18–29 years old, had a high school education or less, and was white were under-immunised for a higher percentage of days than children with parents who were 30 or older (17% vs. 9%, P =.04), had at least some college education (23% vs. 10%, P =.01), and were non-white (13% vs. 5%, P =.04). In comparison to non-Asian children, Asian children had a lower mean percentage of days under-immunized (2% vs. 12%, P =.03). Other parent socio-demographic factors (marital status, income, and first-born status) were not linked to the mean percentage of days under-immunised. | **Brief description of method and analytical process:**  Study was cross-sectional in nature. Data analysis was done with Stata 10 (Stata Corp, College Station, TX).  **Key outcomes relevant to this review**:  Prevalence rates of parental childhood vaccine hesitancy:  For reasons other than illness or allergy, 27% of parents reported delaying and 16% reported refusing a recommended vaccine. More than half (53%) believed that their child would benefit from receiving fewer immunisations at the same time, were afraid that their child would experience an adverse effect from a vaccine (64%) and were worried that childhood vaccines might not be safe (58%).  Predictors of childhood vaccine uptake or intention (p-value < .05):  Parents with at least some college education, mothers, and parents with a household income of more than $75,000 were less likely to agree that getting sick is a better way for their child to develop immunity than getting a shot than parents with a high school education or less and parents with a household income of $75,000, respectively. Parents with at least some college education, on the other hand, were more likely to be concerned that any of the childhood vaccinations might be unsafe. Mothers and parents with a household income of more than $75,000 were more likely to be unconcerned about their child suffering a serious side effect from a vaccination.  Parents of black children were more likely than non-black parents to distrust their child's paediatrician. Parents who were married or living with a partner, as well as those who were 30 years old or older, were less likely to have doubts about their child's doctor. There was no statistically significant difference in socioeconomic characteristics between parents who refused or delayed a recommended childhood immunisation for reasons other than illness or allergy and those who did not.  **How the outcomes were measured:**  Multivariate linear regression models, Factor analysis, Cronbach’s α  **Potential confounders:**  NR  **Ethical issues:**  The Human Subjects Review Committee of Group Health Research Institute approved the study protocol. | **Limitations identified by author:**  First, given the low response rate, the results may reflect response bias. Second, the sample population may not be representative, since many were white married mothers with at least a high school diploma and a household income of more than $50,000. Third, by polling parents about their immunisation attitudes, beliefs, and behaviours after the timeframe in which they were making immunisation decisions (i.e.. after their child turned 19 months rather than during the first 19 months), the survey results may reflect current immunisation perceptions rather than perceptions at the time they were making immunisation decisions. It is difficult to tell if the link between a child's immunisation status and their parent's PACV responses represents their current or previous immunisation attitudes or beliefs because immunisation perceptions can change over time.  **Evidence gaps and/or recommendations for future research:**  NR  **Source of funding:**  Seattle Children’s Centre for Clinical and Translational Research Paediatric Pilot Fund Award |
| 29. | **Researcher(s):**  Opel et al., 2013  **Title:**  The Relationship Between Parent Attitudes About Childhood Vaccines Survey Scores and Future Child Immunization Status: A Validation Study  **Year:**  2013  **Journal:**  JAMA Paediatrics  **Volume:**  167  **Country:**  USA  **WHO Region:**  AMR  **Quality Assessment:**  Include | **What was/were the research question(s):**  What is the predictive validity and test-retest reliability of the Parent Attitudes About Childhood Vaccines survey (PACV)?  **What theoretical approach (e.g., Grounded Theory) does the study take (if specified):**  Self-administered PACV questionnaire (5-7 minutes) | **What population were the sample recruited from:**  English-speaking parents of children born between July 10 and December 10, 2010, and who were members of a Seattle-based integrated health care delivery system.  **How were they recruited:**  Random sampling  **How many participants were recruited:**  310 completed the baseline PACV survey, 220 were involved in the follow-up survey  **Were there specific inclusion criteria:**  English-speaking parents of 2-month-old children born between July 10 and December 10, 2010, and who were part of a large, integrated US health-care delivery system (Group Health Cooperative [GHC], Seattle)  **Were there specific exclusion criteria:**  NR  **Were there specific vaccines under consideration:**  NR  **Other details:**  There was a higher number of Hispanic or Latino parents and lower mean PACV scores among those who stayed continuously enrolled. The demographics of continuously enrolled respondents and test-retest respondents showed no significant variations. | **Brief description of method and analytical process:**  Prospective cohort study. Data analysed using Pearson χ2 tests, Fisher test, McNemer test, Multivariate linear regression models.  **Key outcomes relevant to this review**:  Prevalence rates of parental childhood vaccine hesitancy:  30.4% were very or somewhat hesitant about childhood vaccinations, 23.9% delayed a vaccination for their child for reasons other than illness or allergy, and 7.7% decided not to have their child receive a vaccination for reasons other than illness or allergy.  Predictors of childhood vaccine uptake or intention (p-value < .05):  More than half of parents were concerned that a vaccination may cause a major adverse reaction in their child (57.7%) or that any of the childhood vaccinations may not be safe (51.5%).  **How the outcomes were measured:**  PACV survey tool, Pearson χ2 tests, Fisher test, McNemer test, Multivariate linear regression models.  **Potential confounders:**  NR  **Ethical issues:**  The Group Health Human Subjects Review Committee approved the study protocol. | **Limitations identified by author:**  Given the low response rate, one of the study's limitations was the possibility of response bias. Second, the PACV was not given to the full cohort at the same time, which could have resulted in sampling variance. Third, the study sample set comprised parents who worked for a Seattle-based health-care delivery system. As a result, the findings may not be applicable to different situations or geographic areas. Fourth, most of the respondents in the sample were white, married mothers with a household income of more than $75,000. Although the study population in race/ethnicity and household income closely mirrored the larger GHC population and the 2011 King County population in race/ethnicity, this homogeneity may have hampered the researchers' ability to detect differences in PACV scores or days underimmunized by these characteristics. Finally, the 3-tier PACV categorization was done after the fact. Because of the timing, the classification may be prone to overfitting the relationship between the PACV score and days underimmunised, as well as a lack of reproducibility. This categorisation, as well as the associated results, need validation in other samples.  **Evidence gaps and/or recommendations for future research:**  Validation in other settings or geographic areas  **Source of funding:**  The Centre for Clinical and Translational Research Mentored Scholar Program, Seattle Children’s Research Institute funded the study. |
| 30. | **Researcher(s):**  Rachel et al., 2017  **Title:**  Prevalence of Vaccine Hesitancy Among Expectant Mothers in Houston, Texas  **Year:**  2017  **Journal:**  Academic Paediatrics  **Volume:**  18  **Country:**  USA  **WHO Region:**  AMR  **Quality Assessment:**  Include | **What was/were the research question(s):**  What is the prevalence of vaccine hesitancy among expectant mothers?  **What theoretical approach (e.g., Grounded Theory) does the study take (if specified):**  Self-administered PACV survey (modified for use with expectant mothers attending a single obstetric practice in Houston, Texas) | **What population were the sample recruited from:**  Women who, between July 2014 and September 2015, were between 12 and 31 weeks pregnant and received care at Baylor College of Medicine Obstetrics and Gynaecology practice at the Texas Children’s Pavilion for Women  **How were they recruited:**  Convenience sampling  **How many participants were recruited:**  648  **Were there specific inclusion criteria:**  1) Participants spoke English, 2) The pregnant parent was between 12 and 31 weeks pregnant, 3) Both parents were 18 years old or older, and 4) Neither parent had previously participated in a vaccine study at the study institution.  **Were there specific exclusion criteria:**  NR  **Were there specific vaccines under consideration:**  NR  **Other details:**  Most participants were non-Hispanic white pregnant women who were 30 years old or older and married. About half of the mothers were expecting their first child, and most of them did not perceive their pregnancy to be high-risk. | **Brief description of method and analytical process:**  Cross-sectional study. Data analysed using univariate and multivariate linear regression models.  **Key outcomes relevant to this review**:  Prevalence rates of parental childhood vaccine hesitancy:  8.2% of the mothers were vaccine hesitant  Predictors of childhood vaccine uptake or intention (p-value < .05):  Between hesitant and nonhesitant expectant moms, there was no significant difference in the proportion of first-born children, age group, marital status, household income, number of children in the family, race, or high-risk pregnancies (P >.10).  **How the outcomes were measured:**  PACV scale (modified for use with expectant mothers)  **Potential confounders:**  NR  **Ethical issues:**  Study was approved by the institutional review board of Baylor College of Medicine. | **Limitations identified by author:**  First, the researchers used convenience sampling to recruit participants and missed 46% (n=660) of them due to short waiting room times, which could have resulted in response bias.  Second, they were unable to link 10 expectant fathers with their expectant partners, reducing the size of the ample. Third, the PACV survey that was modified for use with pregnant mothers may not have kept the construct and predictive validity that was observed when it was given to parents. Fourth, the PACV is a generic instrument for assessing vaccine hesitancy that uses 15 validated items across three domains to assess parental vaccine decision-making. It does not specify whether any vaccines are of primary interest to the respondents, nor does it cover all possible vaccine concerns. As a result, before giving targeted vaccine advice to VHPs identified by the PACV, further information from the parents may be required. Finally, because enrolled parents reported a high income and high educational attainment, the study cohort may not be generalizable to other communities or representative of people of the greater Houston area. Furthermore, the participants in this study were all parents who were having treatment at a large medical facility. Vaccine hesitancy may vary among parents who obtain obstetric care from various types of providers, including private practise physicians and midwives.  **Evidence gaps and/or recommendations for future research:**  Future research should consider more representative samples in the Houston area as well as in other settings or geographic regions.  **Source of funding:**  NR |
| 31. | **Researcher(s):**  Stefanoff et al., 2010  **Title:**  Tracking parental attitudes on vaccination across European countries: The Vaccine Safety, Attitudes, Training and Communication Project (VACSATC)  **Year:**  2010  **Journal:**  Vaccine  **Volume:**  28  **Country:**  Multi-national  **WHO Region:**  Multi-regional  **Quality Assessment:**  Include | **What was/were the research question(s):**  What are parental attitudes on vaccinations across countries in Europe?  **What theoretical approach (e.g., Grounded Theory) does the study take (if specified):**  Cross-sectional surveys were used by the Vaccine Safety, Attitudes, Training, and Communication Project (VACSATC) to track parental attitudes toward vaccinations in different European countries. | **What population were the sample recruited from:**  Parents in England, Norway, Poland, Spain, and Sweden with children under the age of three  **How were they recruited:**  Stratified/Random sampling  **How many participants were recruited:**  6611  **Were there specific inclusion criteria:**  NR  **Were there specific exclusion criteria:**  NR  **Were there specific vaccines under consideration:**  NR  **Other details:**  English parents (88%) cited MMR as the vaccine they were most worried about. In comparison, 66% of Swedish doubters, 57% of Norwegian doubters, and only 7% of Polish doubters expressed concerns about the MMR vaccine (the type of vaccine that prompted questions among sceptics was not surveyed in Spain). Polish doubters were more concerned about pneumococci vaccinations (55%) than the MMR vaccine (7%). A third of the English doubters expressed reservations about the combined pentavalent vaccine, which contains DTaP, IPV, and Hib. | **Brief description of method and analytical process:**  Cross-sectional studies were conducted. Country-specific bivariate analyses were done. Because of the diverse sampling and measuring methods used, the study did not include pooled results analysis or computation of confidence intervals.  **Key outcomes relevant to this review**:  Prevalence rates of parental childhood vaccine hesitancy:  A sizable proportion of respondents had reservations regarding vaccination. Parents in England were the most doubtful (28%), followed by Poles and Norwegians (about 20%), and parents in Sweden and Spain (17% and 12% respectively).  Predictors of childhood vaccine uptake or intention (p-value < .05):  Fear of adverse events following vaccination (12% in Spain; 14% in England; 40% in Sweden; 76% in Norway), the alleged link between autism and MMR vaccination (22% in England; 30% in Sweden), vaccine safety and its long-term effects (England 40%; Norway 40%) were the most common reasons for doubt among the doubters.  In Norway, doubters were also concerned because they believed there was some debate among experts on vaccine safety (31%), while in Spain, 24% of doubters said their doubt stemmed from a lack of information regarding vaccination in general. In Poland, the grounds for vaccination hesitancy were not investigated.  **How the outcomes were measured:**  All the studies used questionnaires from the Department of Health England's attitudinal survey. Bivariate analyses were done in all countries.  **Potential confounders:**  NR  **Ethical issues:**  NR | **Limitations identified by author:**  Non-response bias, sample representativeness  **Evidence gaps and/or recommendations for future research:**  Detailed information on non-responders should be collected in future cross-country comparisons of parental attitudes on vaccinations. Researchers should continue the work of standardising attitudinal surveys, both to allow for comparison of clearly defined indicators and to identify shifts in parental perceptions and beliefs that could lead to non-compliance with recommended childhood immunisations.  **Source of funding:**  Directorate-General for Health and Consumers (DG SANCO) |
| 32. | **Researcher(s):**  Strelitz et al., 2015  **Title:**  Parental vaccine hesitancy and acceptance of seasonal influenza vaccine in the paediatric emergency department  **Year:**  2015  **Journal:**  Vaccine  **Volume:**  33  **Country:**  USA  **WHO Region:**  AMR  **Quality Assessment:**  Include | **What was/were the research question(s):**  1) What is the feasibility of administering the PACV modified for influenza vaccination in the Paediatric Emergency Department (PED) setting?  2) Are parental PACV scores associated with patient receipt of influenza vaccine in the PED?  **What theoretical approach (e.g., Grounded Theory) does the study take (if specified):**  PACV survey tool (modified for influenza vaccination) (5-7 minutes response time) | **What population were the sample recruited from:**  Parents attending the PED of an urban, tertiary paediatric hospital in Seattle, Washington  **How were they recruited:**  Convenience sampling  **How many participants were recruited:**  152  **Were there specific inclusion criteria:**  1) English-speaking parents of children aged 6 months to 7 years, 2) Parents who were afebrile and medically stable enough to be discharged from the PED, and 3) Parents who had not yet received an influenza vaccine that season.  **Were there specific exclusion criteria:**  1) Non-English-speaking parents, and 2) Parents of children older than 7 years.  **Were there specific vaccines under consideration:**  Influenza vaccine  **Other details:**  Most of the participants were non-Hispanic White (53%), over 30 years old (68%), married (71%), had some college degree or higher education (75%), and had less than or equal to $75,000 (65%) in household income; just under half of the children had public insurance (49%). | **Brief description of method and analytical process:**  Cross-sectional study in the PED of a tertiary paediatric hospital in Seattle, Washington during the 2013–2014 influenza season. Multivariate linear regression models were used for analysis.  **Key outcomes relevant to this review**:  Prevalence rates of parental childhood vaccine hesitancy:  26% of parents were found to be vaccine hesitant (PACV score ≥ 50). 63% of this hesitant sample refused the influenza vaccine when it was offered in the PED.  Predictors of childhood vaccine uptake or intention (p-value < .05):  When compared to parents with more than a high school education, parents with a high school education or less had a lower likelihood of refusing the influenza vaccine (odds ratio [OR] 0.4, 95% CI: 0.2–0.9). When compared to parents of children with lower acuity ratings, parents of children with higher acuity ratings had a higher likelihood of refusing the influenza vaccine (OR 2.0, 95% CI: 1.2–3.6). In addition, Hispanic parents were less likely than non-Hispanic White parents to refuse the influenza vaccine (OR 0.2, 95% CI: 0.1–0.6).  **How the outcomes were measured:**  PACV scale, Multivariate logistic regression models.  **Potential confounders:**  NR  **Ethical issues:**  The Western Institutional Review Board approved the study protocol. | **Limitations identified by author:**  Because only parents who presented to a single PED were recruited, the data may not be generalisable. The study was also prone to selection bias due to the convenience sampling of parents, as well as sampling bias due to study personnel not being blinded to the parents' vaccination decision. Finally, the study was limited by enrolling participants for five months in a row during a single influenza season. Furthermore, researchers began enrolling participants in November, even though the seasonal influenza vaccine had been available since September. A greater proportion of non-hesitant (vs. hesitant) parents may have already vaccinated their children by the start of the study, making them ineligible to participate. Furthermore, as the season progressed, parents may have been more inclined to refuse the influenza vaccine merely because the perceived benefit of the vaccine was lower than it had been earlier in the season.  **Evidence gaps and/or recommendations for future research:**  More research is required to determine the impact of administering the PACV in the PED on physician behaviour and parental vaccine uptake.  **Source of funding:**  1) Translational Research Ignition Projects Program of the Seattle Children’s Research Institute Centre for Clinical and Translational Research  2) The National Centre for Advancing Translational Sciences of the National Institutes of Health. |
| 33. | **Researcher(s):**  Ucakar et al., 2018  **Title:**  Vaccine confidence among mothers of young children, Slovenia, 2016  **Year:**  2018  **Journal:**  Vaccine  **Volume:**  36  **Country:**  Slovenia  **WHO Region:**  EUR  **Quality Assessment:**  Include | **What was/were the research question(s):**  What is the vaccine confidence level among mothers of young children in Slovenia?  **What theoretical approach (e.g., Grounded Theory) does the study take (if specified):**  Self-administered questionnaire developed for the study | **What population were the sample recruited from:**  Mothers who gave birth in 2014–15 (sampled from the national perinatal information system)  **How were they recruited:**  Simple random sampling  **How many participants were recruited:**  1704  **Were there specific inclusion criteria:**  Women who gave birth in the years 2014–15 and were recorded in the Perinatal Information System of the Republic of Slovenia database (a medical registry collecting data on all deliveries and births in Slovenia).  **Were there specific exclusion criteria:**  Having a stillborn child, giving birth numerous times during the stipulated term, citing a place of residence outside Slovenia, or not having a reported place of residence  **Were there specific vaccines under consideration:**  NR  **Other details:**  51.9% of mothers who were not at all confident in immunisations cited friends as reliable sources of information. | **Brief description of method and analytical process:**  Cross-sectional study. Data analysis was done using STATA package version 12.1 (Stata Statistical Software: release 12.1 College Station. TX: Stata Corporation) and R (A language and environment for statistical computing. R Foundation for Statistical Computing. Vienna, Austria).  **Key outcomes relevant to this review**:  Prevalence rates of parental childhood vaccine hesitancy:  34.2% were undecided about vaccines  Predictors of childhood vaccine uptake or intention (p-value < .05):  Vaccine confidence was linked to confidence in the health system and confidence in a child's paediatrician. No significant differences were observed in the number of mothers who were vaccine confident across the country or according to other variables such as marital status, social class, or level of education. Except for age, there was no significant differences between the socio-demographic parameters and vaccine confidence. Older mothers were more likely to be vaccine confident than younger mothers.  **How the outcomes were measured:**  Survey instrument, Spearman’s rank correlation coefficient  **Potential confounders:**  NR  **Ethical issues:**  Study protocol was approved by the Republic of Slovenia National Medical Ethics Committee | **Limitations identified by author:**  Because of the survey's simple random sampling technique, there was a risk of selection bias, as more women with specific vaccine sentiments (positive or negative) were more or less likely to answer. Due to this bias, vaccine confidence may have been overestimated or underestimated. The study only included mothers of young children, therefore generalising the findings to all parents in Slovenia should be done with caution. Another limitation of this study is that the questions were not vaccine specific, therefore mothers were unable to express their confidence in specific vaccines.  **Evidence gaps and/or recommendations for future research:**  Because vaccine confidence may be linked to behaviour, the disparity between the low proportion of vaccine confident mothers and Slovenia's comparatively high vaccination coverage merits additional investigation. More research is needed to see how this overwhelmingly negative attitude toward vaccination among Slovenian mothers translates into a reduction in childhood vaccination coverage.  **Source of funding:**  1) Slovenian Research Agency  2) Slovenian Ministry of Health |
| 34. | **Researcher(s):**  Wallace et al., 2019  **Title:**  Development of a valid and reliable scale to assess parents’ beliefs and attitudes about childhood vaccines and their association with vaccination uptake and delay in Ghana  **Year:**  2019  **Journal:**  Vaccine  **Volume:**  37  **Country:**  Ghana  **WHO Region:**  AFR  **Quality Assessment:**  Include | **What was/were the research question(s):**  1) Developing a validated scale to measure parents’ attitudes towards vaccinations in low and middle-income countries  2) What is the proportion of vaccine confident mothers from Northern Region of Ghana?  **What theoretical approach (e.g., Grounded Theory) does the study take (if specified):** | **What population were the sample recruited from:**  Ghanaian parents of children aged 12– 35 months  **How were they recruited:**  Random sampling  **How many participants were recruited:**  373  **Were there specific inclusion criteria:**  NR  **Were there specific exclusion criteria:**  NR  **Were there specific vaccines under consideration:**  DTP, oral polio, rotavirus, MMR, pneumococcal conjugate  **Other details:**  A significant minority (23%) believed that healthy children did not require immunizations and expressed concerns about the number of vaccinations given, with 41% agreeing that children receive more vaccinations than they need and 23% disagreeing that children should receive two injectable vaccinations in one visit rather than one per visit. | **Brief description of method and analytical process:**  The study was cross-sectional in nature. The researchers employed exploratory factor analysis (EFA) to determine the validity of the CVAS content, whereas parallel analysis was performed to determine the number of factors to extract. Factor extraction was done using principal axis factor analysis. The scale's reliability was determined using McDonald's Omega coefficient.  **Key outcomes relevant to this review**:  Prevalence rates of parental childhood vaccine hesitancy:  22% were vaccine hesitant while 15% were vaccine refusers  Predictors of childhood vaccine uptake or intention (p-value < .05):  Children of non-schooling mothers were less likely than children of school-going mothers to receive the third dose of diphtheria-tetanus-pertusis-containing vaccine (DTPcv3) (88% versus 95% respectively). Similarly, children of Traditionalist parents were less likely to have received DTPcv3 than children of Christian and Muslim parents (81% versus 92% and 90%, respectively). Firstborn children were slightly more likely than later-born children to have received DTPcv3 (93% versus 90%), as were female children compared to male children (93% versus 88%).  **How the outcomes were measured:**  Caregiver vaccination attitudes scale (CVAS), Exploratory factor analysis (EFA), McDonald’s Omega coefficient, Generalised estimating equation (GEE) models  **Potential confounders:**  NR  **Ethical issues:**  Study protocol was approved by the Ghana Health Services ethics review board, the CDC Human Subjects Office, and the Emory University Institutional Review Board | **Limitations identified by author:**  Because the survey was cross-sectional, the researchers collected data for both the scale validation and vaccination status at the same time; thus, the criterion validity was restricted to concurrent rather than predictive validity. The sample size was adequate for EFA, but the team were unable to divide the sample into two sets to perform confirmatory factor analysis, which could have further strengthened their findings.  **Evidence gaps and/or recommendations for future research:**  Continued development of the scale and validation in other settings and geographical areas  **Source of funding:**  The United States Centres for Disease Control and Prevention (CDC) |
